# Injury triggers fascia fibroblast collective cell migration to drive scar formation through N-cadherin

**DOI:** 10.1038/s41467-020-19425-1

**Published:** 2020-11-06

**Authors:** Dongsheng Jiang, Simon Christ, Donovan Correa-Gallegos, Pushkar Ramesh, Shruthi Kalgudde Gopal, Juliane Wannemacher, Christoph H. Mayr, Valerio Lupperger, Qing Yu, Haifeng Ye, Martin Mück-Häusl, Vijayanand Rajendran, Li Wan, Juan Liu, Ursula Mirastschijski, Thomas Volz, Carsten Marr, Herbert B. Schiller, Yuval Rinkevich

**Affiliations:** 1 Helmholtz Zentrum München, Institute of Lung Biology and Disease, Group Regenerative Biology and Medicine, Munich, Germany; 2 Helmholtz Zentrum München, Institute of Lung Biology and Disease, Group Systems Medicine of Chronic Lung Disease, Munich, Germany; 3 Helmholtz Zentrum München, Institute of Computational Biology, Munich, Germany; 4 Mira-Beau gender esthetics, Berlin, Germany; 5 Wound Repair Unit, CBIB, Faculty of Biology and Biochemistry, University of Bremen, Bremen, Germany; 6 Department of Dermatology and Allergology, Technical University of Munich, School of Medicine, Klinikum rechts der Isar, Munich, Germany; 7 German Centre for Lung Research (DZL), Munich, Germany

**Keywords:** Skin models, Time-lapse imaging, Cadherins

## Abstract

Scars are more severe when the subcutaneous fascia beneath the dermis is injured upon surgical or traumatic wounding. Here, we present a detailed analysis of fascia cell mobilisation by using deep tissue intravital live imaging of acute surgical wounds, fibroblast lineage-specific transgenic mice, and skin-fascia explants (scar-like tissue in a dish – SCAD). We observe that injury triggers a swarming-like collective cell migration of fascia fibroblasts that progressively contracts the skin and form scars. Swarming is exclusive to fascia fibroblasts, and requires the upregulation of N-cadherin. Both swarming and N-cadherin expression are absent from fibroblasts in the upper skin layers and the oral mucosa, tissues that repair wounds with minimal scar. Impeding N-cadherin binding inhibits swarming and skin contraction, and leads to reduced scarring in SCADs and in animals. Fibroblast swarming and N-cadherin thus provide therapeutic avenues to curtail fascia mobilisation and pathological fibrotic responses across a range of medical settings.

## Abstract (web summary)

Extensive scars develop in deep wounds as opposed to superficial wounds but it is unclear why. Here, the authors use live imaging of physiologic wounds and scars formed ex vivo to show that fascia fibroblasts upregulate N-cadherin allowing coordinated cell migration that drives extensive scar formation of deep wounds.

## Introduction

Organisms such as planaria, hydra, zebrafish and certain amphibians, are able to fully regenerate tissues and organs upon injury with minimal scarring. However, the global mammalian and human response to injury is scarring and contraction over the wound. Scars replace the original connective tissue foundation with dense “plugs” of matrix fibres that tighten the tissue and reduce its flexibility^1,2^. Scarring occurs both after accidental injuries and after surgery in over 100 million patients per year and particularly among children, one in five of whom require reconstructive surgery for scar-induced contracted joints^2–5^. Scarring and impaired wound-healing are major clinical problems and a tremendous burden for patients and global healthcare systems, costing tens of billions of dollars per year in the US.

Whereas deep skin wounds trigger the subcutaneous fascia to form exuberant scars, there are rare examples where skin injuries culminate in scarless regeneration. Scarless regeneration is prevalent during foetal life as well as in certain anatomic locations such as the oral mucosa^6,7^. The African spiny mouse (genus *Acomys*)^8^ is another rare example of a mammalian species that can regenerate skin wounds without scarring. Despite extensive study into scarring, the underlying mechanism of how scars emerge remains incompletely understood. Understanding this universal wound-repair event would allow us to control, restore and preserve the functions of physically damaged adult tissues and pave the way towards clinically regenerating severe skin injuries.

Recent studies have uncovered fibroblasts with distinct potencies based on their lineage origins in certain skin locations^9–14^. For example, we found that fibroblasts with scar-forming potential coexist with “ordinary” fibroblasts in the adult back-skin^12,13^. Scarring ability of fibroblasts is instilled during embryogenesis in cells with temporary early embryonic expression of the Engrailed-1 gene. In adults the scar-forming and non-scar-forming fibroblasts are thus referred to as the embryonic “engrailed-past” and “engrailed-naive” fibroblasts, EPFs and ENFs, respectively. We recently found that subcutaneous fascia, a viscoelastic membranous sheet of matrix, that creates a thin gelatinous and frictionless interface between the skin and the body’s interior rigid structures, is the main anatomical contributor to exuberant scars that form following deep skin wounds^9^. We have shown that injury induces fascia mobilisation across the skin, and that fascia mobilisation repairs breaches in the structural continuums of the skin, thereby preserving skin integrity and function. It remains unclear why fascia EPFs are prone to form scars, whereas upper dermal or oral mucosa fibroblasts do not.

In this study, by using intravital probing into deep wounds of live mice and an ex vivo explant technique termed scar-like tissue in a dish (SCAD) coupled with genetically traceable scar-forming EPFs, we show that upon wounding fascia EPFs upregulate N-cadherin, which is required for the collective migration and subsequent swarming of fascia EPFs towards wound centre and drives scar formation. Blocking N-cadherin by peptide inhibitor or genetic modification reduces scarring.

## Results

### Collective migration of fascia EPFs in deep wounds

**Fig. 1:**
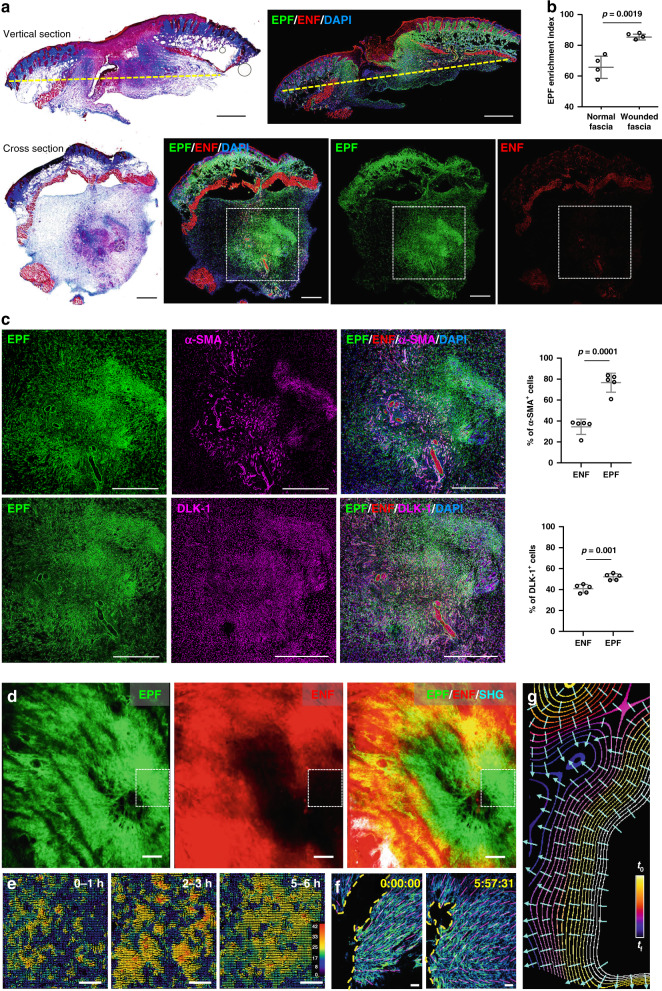
Collective migration of fascia EPFs in physiological in vivo wounds. **a** Masson’s trichrome staining and fluorescence images of vertical sections and transverse cross sections of wounds at 7-dpi from *En1*^Cre^*,R26*^mTmG^ mice. EPFs were shown in green, ENFs in red, nuclei stained with DAPI in blue. The yellow dashed lines in vertical sections indicate the position of the cross sections. **b** EPF enrichment index in uninjured normal fascia or wounded fascia. Mean ± SD, *p* = 0.0019, unpaired two-tailed *t*-test, *n* = 4. **c** Immunolabelling of α-SMA or DLK-1 in magenta of boxed area showed in **a**, and quantification of the percentages of α-SMA^+^ or DLK-1^+^ cells in EPFs and ENFs. Mean ± SD, unpaired two-tailed *t*-test, *p* = 0.0001 (α-SMA), *p* = 0.001 (DLK-1), *n* = 5. **d** 3D image of in vivo wound at 14-dpi on the back of *En1*^Cre^*;R26*^mTmG^ mice. White boxes indicate field of view for intravital live imaging (Supplementary Movie 1) and particle image velocity (PIV) analysis. Images are representative of three biological replicates. **e** PIV analysis of EPF migration over the indicated time. The colour-coded vectors indicate the direction and displacement in pixels. **f** EPF channel of the first and last frames of the intravital recording (Supplementary Movie 2). The yellow dash lines indicate the leading front of invading fibroblasts, purple lines are predicted EPF cell orientation. **g** Contour lines are smoothened EPF leading front in selected frames from the intravital recording. Colours indicate time, bright for the beginning (*t*_0_) and dark for the end (*t*_f_). Cyan lines are predicted trajectories of EPF swarm that are perpendicular to contour lines. Scale bars: **a**, **b**, **d** = 500 µm; **e**, **f** = 50 µm.

First, we looked into the composition of fascia fibroblasts during wound healing and scarring. We previously discovered that scars developed from a specific type of fibroblast that temporarily expressed *Engrailed-1* early in embryogenesis (termed EPFs). Whereas ENFs that have no history of *Engrailed-1* expression, do not contribute to scar formation in any way^12,13^. We followed the fates of these two fibroblast lineages by crossing fibroblast lineage-specific promoter mice (*En1*^Cre^) to mice (*R26*^mTmG^*)* with a dual fluorescence reporter that replaces membrane-bound tdTomato with membrane-bound green fluorescence protein (GFP) upon recombination. In all, 5-mm full-thickness excisional wounds were created on the back of *En1*^Cre^;*R26*^mTmG^ mice with biopsy punches. The excision went through the panniculus carnosus muscle and fascia completely. The wounds were splinted with silicone rings to minimise contraction and to allow healing through granulation and re-epithelialization, similar to human skin wounds. To zoom into subcutaneous fascia, we made transverse cross-section of wounds 7 days after wounding and examined the sections that were surrounded by panniculus carnosus of the adjacent normal skin. EPFs were clearly clustered at the wound centre (Fig. 1a), and were enriched from 65.6 ± 6.2% in uninjured subcutaneous fascia (Fig. 1b and Supplementary Fig. 1) to 85.3 ± 1.7% in day-7 wounds (Fig. 1b). Fascia EPFs that clustered at wound centres were 76.6 and 52.2% positive for the myofibroblast markers α-SMA and DLK-1, respectively (Fig. 1c).

To visualise how fascia EPFs become enriched in late wounds and in scars, we analysed physiological wounds using intravital microscopy coupled with a skin chamber that was placed over a full-thickness back-skin wound *in En1*^Cre^*,R26*^mTmG^ reporter mice. 3D snapshots of the whole in vivo wound at 14 days post-injury (dpi) clearly showed EPFs were enriched in wound centres, where they aggregate into a swarm eye that is devoid of ENFs (Fig. 1d). Supplementary Movie 1 shows a high-resolution 6-h intravital live recording of fascia EPFs at the leading front of the swarm (Supplementary Movie 1). Particle image velocimetry (PIV) analysis revealed that individual foci of EPFs join into large collective swarms that move towards wound centres (Fig. 1e). Prediction measurements of cell orientations (see Methods section) showed that EPFs collectively align themselves towards the leading front as a group (Fig. 1f and Supplementary Movie 2). Furthermore, cell trajectory measurements clearly demonstrated fascia EPFs migrated collectively in physiologic wounds towards the wound centre (Fig. 1g).

### Fascia EPFs swarm to form scars

Intravital live imaging in animals is limited to 6-h intervals, revealing only fragments of scarring event. In order to visualise the entire scarring process, we invented an ex vivo skin-fascia explant culture technique termed as SCAD.

**Fig. 2:**
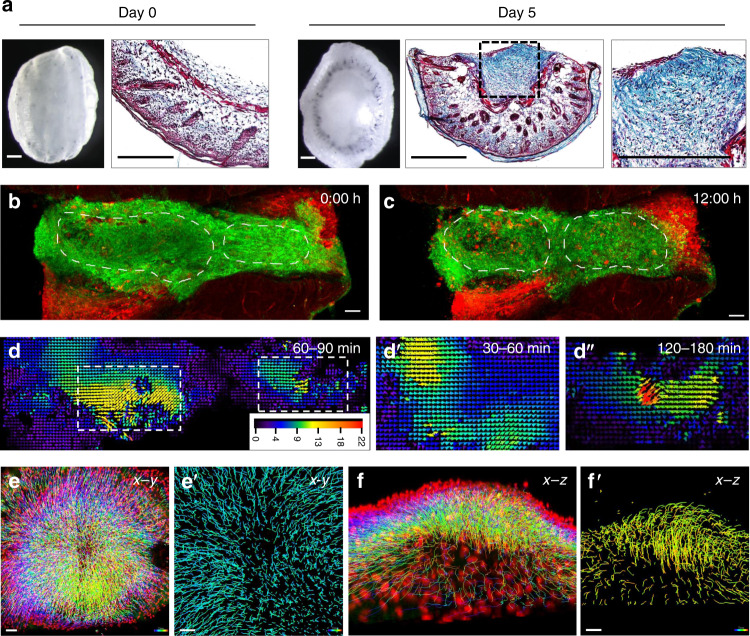
Fascia EPFs swarm during scarring in SCAD. **a** Whole-mount bright-field images and Masson’s trichrome staining with collagen in blue of fresh SCAD (day 0) and after 5-day culture (day 5). *n* > 1000. **b**, **c** Snapshots of live imaging of day 3 *En1*^Cre^*;R26*^mTmG^ SCAD. Two independent EPF aggregates are encircled with a dotted line at time *t* = 0 h (**b**) and *t* = 12 h (**c**) (Supplementary Movie 4). Images are representative of four biological replicates. **d** PIV analysis of GFP channel from live imaging showed in Supplementary Movie 4. Arrowheads indicate the direction of particle movement. Particle velocity is indicated by a scale from slow (blue) to fast (red). Scale bar unit: pixel. **d′**–**d″** Vector map from PIV analysis of the left swarm over 30–60 min (**d′**) and of the right swarm over 120–180 mins (**d″**). **e**, **f** Colour-coded tracking of EPFs from live imaging (72–96 h) of *En1*^Cre^*;R26*^LSL-H2B-mCherry^ SCAD, from top view (**e**) and side view (**f**). Colours indicate time, starting from blue to red at the end of the movie (Supplementary Movie 6). **e′** Enlarged images of EPF migration tracks in the scar centre at the beginning of swarming (blue-to-cyan). **f′** Enlarged images of EPF migration tracks at the end of swarming (green-to-orange). Images are representative of four biological replicates. Scale bars: **a** = 500 µm; **b**, **c**, **e**, **f** = 50 µm; **e′**, **f′** = 30 µm.

In brief, we excised and incubated 2-mm full-thickness mouse skin discs that included the epidermis, dermis, panniculus carnosus and subcutaneous fascia, with the fascia side facing upwards (Supplementary Fig. 2a). Under these conditions, skin-fascia explants developed uniform scars over a course of 5 days (Fig. 2a and Supplementary Fig. 2b). The technique worked with skin grafts of all ages from embryonic day 18.5 to adult (Supplementary Fig. 2c). These were bona fide scars by several different parameters. Histologic sections revealed masses of fibroblasts that developed increasingly woven connective tissue that bridged between skin folds. The fascia fibroblasts created a plug of collagen fibres that progressively formed scars (Fig. 2a, Day 5). 3D-immunolabelling of fibrillar matrix proteins revealed abundant fibronectins and collagen I aligning within ex vivo scars, with no elastin, just like in animals (Supplementary Fig. 3a, b). More conclusively, fractal analysis^12^ of extracellular matrix lattice arrangements indicated that our ex vivo scars are structurally identical to those developing in animals, with similar lattice complexity and porosity values (Supplementary Fig. 3c, d). Mass spectrometry-driven proteomic analysis and subsequent String protein–protein interaction network analysis revealed a similar matrix protein composition and protein–protein interaction between SCAD and in vivo scars (Supplementary Fig. 3e, f and Supplementary Data 1). Unlike reconstructed 3D skin models, SCADs contained most skin and fascia cell types including leucocytes, macrophages, lymph and blood vessels, adipocytes, panniculus carnosus muscle, pigmented melanocytes and β3-tubulin positive nerve bundles, and their native microenvironments at unprecedented resolution during wounding (Supplementary Fig. 4a–g). When SCADs made from *En1*^Cre^;*R26*^mTmG^ mice, where EPFs and ENFs were distinctly labelled green and red respectively, EPFs were the major contributor to scar (Supplementary Fig. 4h, i), and expressed myofibroblast markers α-SMA and DLK-1 (Supplementary Fig. 4j, k), exactly as in animals (Fig. 1a, b, d). α-SMA expression in EPFs was initially low (3.6 ± 2.7%) but substantially increased in SCADs on day 5 (41.3 ± 13.3%) mirroring the upregulation of α-SMA on myofibroblasts in physiologic wounds (Supplementary Fig. 5). Collectively, these data demonstrate that SCADs have scars with identical matrix structure and protein composition, the same diverse cell types, fibrogenic cell origins and identical surface markers to in vivo fascia scars.

Having found that mobilisation of fascia tissue leads to scars in wounds, and can be studied ex vivo, we set out to view how fascia is mobilised and form real scars using multi-photon live imaging microscopy in SCAD.

Fractal analysis of SCADs from genetically labelled mice indicated that fascia EPFs uniquely increase intercellular connectivity within several hours after injury. Certain regions had a simple porous structure, indicative of healthy fascia, whereas other areas developed a complex and smooth architecture, characteristic of an early stage scar in physiologic wounds (Supplementary Fig. 6a–c). In order to view the onset of fascia EPF migration, we generated chimeric SCADs where the interior part of the 4-mm tissue with green-labelled EPFs was replaced with 2-mm red-labelled tissue from a separate transgenic mouse. This mixing of tissues allowed single fascia EPFs to be clearly visualised as they entered the red-only inner tissue. The fascia EPFs clearly invaded the inner circle as aggregates within 24 h and each aggregate of EPFs moved collectively as a group (Supplementary Fig. 6d and Supplementary Movie 3).

By day 3, we frequently observed several distinct swarm clusters varying in size within a single SCAD. At least 4 swarms could be seen in Supplementary Movie 4. Two large swarms from SCADs were outlined at the 72 h and 96 h of the scarring process in Fig. 2b, c. Individual EPF swarms had various speeds and they changed direction regularly with a periodicity between one to two hours (Fig. 2d–d″).

To map the overall cell trajectory of all the EPF swarms, we wanted to move from manual to computational cell tracking, but the highly variable shapes of fibroblasts makes this difficult. We therefore crossed our fibroblast lineage-specific promoter mice to a nuclear mCherry reporter line and imaged SCADs from the offspring. Strikingly, all fascia EPFs coalesced into a single mega-swarm by day 4 (Fig. 2e, f). Fascia EPFs at the periphery moved towards the centre (Fig. 2e, e′). Once at the centre, fascia EPFs migrated vertically and towards the epidermis, generating a swarm eye of densely packed EPFs that contracted the skin from all sides (Fig. 2f, f′). The top view in Supplementary Movie 5 showed the collective movements of EPFs (mCherry-positive nuclei), as a single swarm across the dermis and the side view in Supplementary Movie 6 showed migratory tracks of EPFs towards epidermis.

**Fig. 3:**
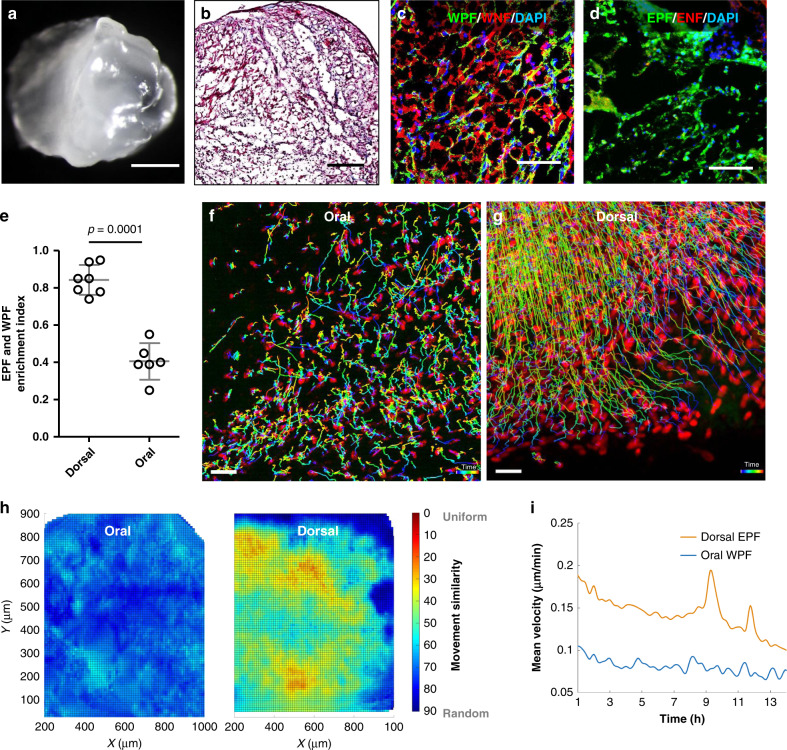
Fibroblast swarms are absent from oral mucosa SCAD. **a**, **b** Whole-mount bright-field image (**a**) and Masson’s trichrome staining (**b**) of SCAD from buccal mucosa. *n* > 30. **c**, **d** Fluorescence images of SCAD from *Wnt1*^Cre^*;R26*^mTmG^ buccal mucosa (**c**) and *En1*^Cre^*;R26*^mTmG^ back-skin (**d**). **e** EPF and WPF enrichment index in day 5 *En1*^Cre^*;R26*^mTmG^ dorsal SCAD or *Wnt1*^Cre^*;R26*^mTmG^ oral SCAD, respectively. Mean ± SD, unpaired two-tailed *t*-test, *p* = 0.0001, *n* = 7/6. **f**, **g** Colour-coded tracking of WPFs in oral SCAD (**f**, Supplementary Movie 8) or tracking of EPFs in dorsal SCAD (**g**) on a mCherry nuclear reporter (72–96 h). The colour indicates the time, with purple at the beginning and red at the end of the movie. Images are representative of three biological replicates. **h** Movement similarity between WPF and EPF tracks is visualised in a scale from 0 (red, uniform migration) to 90 (blue, random migration). **i** Comparison of velocities of WPFs in oral SCADs (blue) versus fascia EPFs in dorsal SCAD (orange) over time. Lines shown are smoothing lines over all velocity values at each time point. Scale bars: **a** = 500 µm; **b** = 200 µm; **c**, **d** = 50 µm; **f**, **g** = 30 µm.

Having discovered fibroblast swarms during back-skin scarring, we went on to investigate if swarms occur in the oral mucosa, an anatomic region that lacks fascia tissue and heals with minimal scarring, unlike the back-skin. We used a reporter mouse that permanently labels Wnt1-lineage positive fibroblasts (WPFs) that heal wounds in cranial skin and oral mucosa^7,13^. We crossed fibroblast lineage-specific reporter mice (*Wnt1*^Cre^) to *R26*^mTmG^ mice and made SCADs from the buccal mucosa of double-transgenic offspring (*Wnt1*^Cre^*;R26*^mTmG^). These tissues had no scars and minimal bundles of aligned collagen (Fig. 3a, b).

WPFs remained intermixed with other fibroblasts at all time and did not form pre-scar aggregates, compared to EPFs in back-skin SCADs (Fig. 3c–e). WPFs remained at ~50% in oral SCADs throughout the 5 days (Fig. 3e), similar to healthy oral mucosa.

To analyse the complete migration dynamics of WPFs at the whole-tissue level, we crossed *Wnt1*^Cre^ and nuclear mCherry reporter mice and made SCADs from buccal mucosa of the offspring. The migration and computational tracking of WPFs as showed in Supplementary Movies 7 and 8 respectively, revealed that oral mucosa fibroblasts moved stochastically without migrating in any particular direction, i.e., completely unlike fascia EPFs from back-skin (Fig. 3f–h). Oral mucosa fibroblasts migrated at around half the speed of back-skin fascia EPFs without the periodic shrivelling and relaxation seen in back-skin (Fig. 3i). Oral mucosa fibroblasts also did not swarm together (Supplementary Movie 9) and did not generate the same tensile forces and tissue bending movements as back-skin fascia EPFs. Thus, the swarming that causes scarring is an exclusive feature of the scar-forming fascia EPFs.

Our in vivo and ex vivo imaging and analyses revealed a collective migration pattern of fascia EPFs in wounds that build in a crescendo, with individual foci of EPFs joining into large collective swarms. Overall the collective swarms were remarkably similar when viewed by intravital imaging deep in mice and when viewed in ex vivo SCAD cultures.

### Fibroblast swarms are driven by N-cadherin

Since fascia EPFs swarm during scar formation, we reasoned that they must require an adhesion molecule to aggregate. Cell adhesion is a calcium-dependent process. We therefore tested our hypothesis by inhibiting cell–cell adhesion in SCADs with the Ca^2+^ chelator EGTA. The blockage of Ca^2+^ signalling resulted in significantly reduced scarring (Supplementary Fig. 7a).

**Fig. 4:**
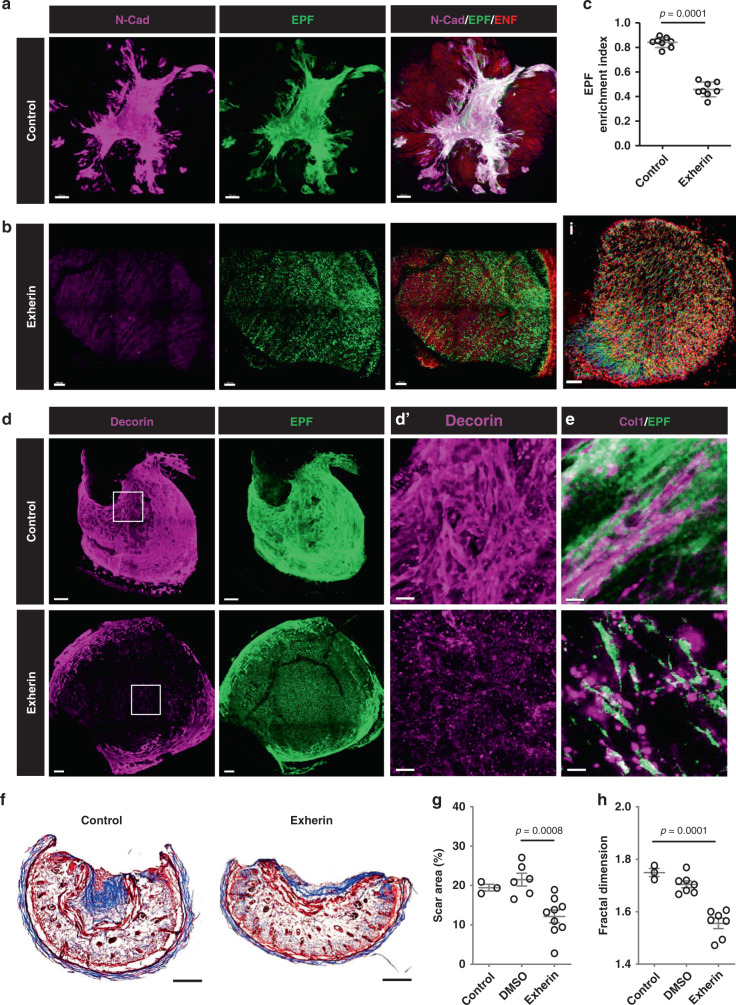
Fibroblast swarms are driven by N-cadherin. 3D immunolabelling of N-cadherin (magenta) in control (**a**) or 500 µg/ml Exherin treated (**b**) *En1*^Cre^;*R26*^mTmG^ SCAD. **c** EPF enrichment index in control or Exherin-treated SCAD. Mean ± SD, *p* = 0.0001, unpaired two-tailed *t*-test, *n* = 8. 3D immunolabelling of decorin (**d**) or collagen I (**e**) in a control (upper panel) or Exherin-treated (lower panel, Supplementary Movie 10) SCAD. **d′** High magnification of white box in **d**. **f** Masson’s trichrome staining of control (upper) or Exherin-treated (lower) SCAD. **g** Scar areas of control, or DMSO treated or Exherin-treated SCADs. One-way ANOVA Tukey’s test, *p* = 0.0008, *n* = 3/6/9. **h** Fractal dimension analysis of control, or DMSO treated or Exherin-treated SCADs. One-way ANOVA Tukey’s test, *p* = 0.0001, *n* = 3/7/7. **i** Colour-coded tracking of fascia EPFs from live imaging (72–96 h) of 500 µg/ml Exherin-treated *En1*^Cre^*;R26*^LSL-H2B-mCherry^ SCAD. Colours indicate time, starting from blue to red at the end of the movie (Supplementary Movie 11). Scale bars: **a**, **b**, **d**, **i** = 100 µm; **d′** = 20 µm; **e** = 50 µm.

To determine which specific adhesion molecule was involved in swarming, we tested a panel of candidates for co-localisation with fascia EPFs in the scar area and found a clear co-localisation of N-cadherin (Fig. 4a). Among other adhesion molecules, notably integrins failed to co-localise (Supplementary Fig. 7b–d). However, the downstream effector of N-cadherin, α-catenin, did co-localise with fascia EPFs and N-cadherin in the scar area, consistent with a potential role for this molecule in swarming and scarring (Supplementary Fig. 7e). N-cadherin was minimally expressed by fascia EPFs upon wounding and was upregulated during the healing and scarring process (Fig. 4a and Supplementary Fig. 7f, g).

In contrast, WPFs in oral SCADs minimally expressed N-cadherin (Supplementary Fig. 7h). We therefore specifically blocked N-cadherin in *En1*^Cre^*;R26*^mTmG^ SCADs with 500 µg/ml Exherin, a synthetic peptide that selectively inhibits N-cadherin^15,16^. Under Exherin treatment, EPFs refrained from aggregating and remained dispersed in the fascia and skin bending and folding was completely inhibited (Fig. 4b, c). Exherin treatment also abolished the wound-induced upregulation of α-SMA expression in fascia EPFs (Supplementary Fig. 5). Furthermore, the Exherin-treated tissues had profoundly altered expression pattern of decorin (Fig. 4d, d′), a marker of scar matrix assembly that binds to collagen fibrils^17^. Indeed, we observed that non-treated tissues had densely packed fascia EPFs with organised collagen I fibres, whereas Exherin-treated tissues had a mixed “salt and pepper” pattern of granular collagen deposition without uniform fibre bundles (Fig. 4e). Supplementary Movie 10 demonstrated this “salt and pepper” pattern of collagen was throughout the entire Exherin-treated SCAD. Correspondingly, Exherin-treated SCAD showed minimal scar by histology (Fig. 4f, g) and distinct collagen fibre arrangements to control scars by fractal analysis (Fig. 4h). These specific effects of Exherin were not due to increased cell death (Supplementary Fig. 7i, j) or reduced proliferation of EPFs (Supplementary Fig. 8a–c). In fact, the cell proliferation was not essential for the initial stage of scar formation (Supplementary Fig. 8d).

Compared to the mega-swarms and centripetal movements seen in control SCADs and in animals (Fig. 2e and Supplementary Movie 5), Exherin-treated fascia EPFs had greatly diminished collective migration (Fig. 4i and Supplementary Movie 11).

Overall, these data demonstrate unequivocally that N-cadherin facilitates the fibroblast swarming that leads to and links fascia mobilisation in wounds with scarring.

### N-cadherin drives scarring in animals and in human skin

To test if our mechanistic findings from SCADs occur in animals, we first generated full-thickness wounds on backs of animals and found that the dynamic upregulation of N-cadherin in in vivo wounds mimics N-cadherin expression found in SCADs, with minimal expression in healthy skin and significantly elevated expression in day-7 and day-14 wounds, which co-localised with EPFs in scars (Supplementary Fig. 9).

**Fig. 5:**
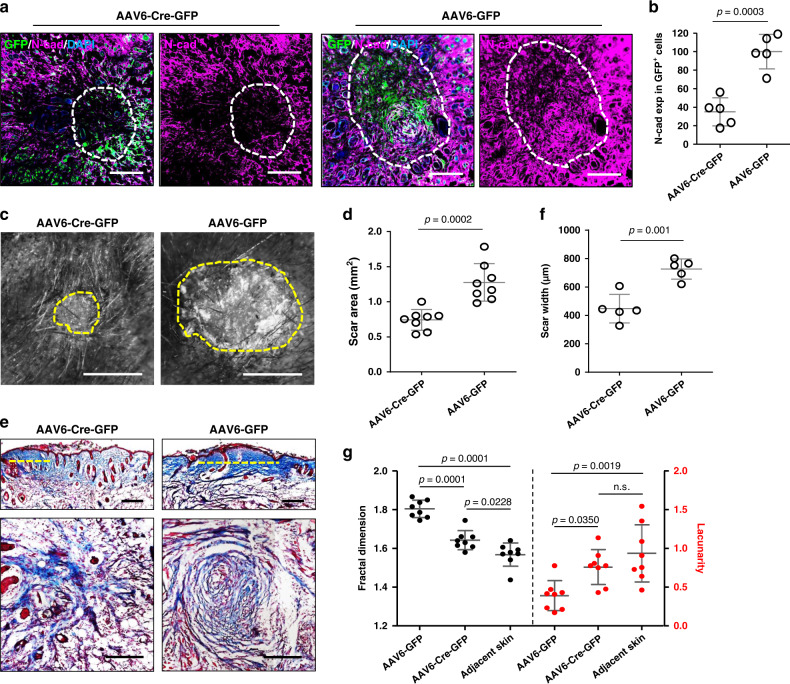
N-cadherin is crucial for scar formation in vivo. N-cadherin was locally knockout around wounds on *Ncad*^fl/lfl^ mice by injection of Cre-expressing AAV6-Cre-GFP virus. AAV6-GFP virus served as control. **a** Immunolabelling of N-cadherin on transverse cross-sections of harvested scars on 14-dpi. GFP indicates transduced cells. Dash lines outline the scar edges. **b** N-cadherin expression in GFP^+^ cells based on immunofluorescence analysis. Data are normalised on the mean of N-cadherin expression in AAV6-GFP wounds. Mean ± SD, *n* = 5, *p* = 0.0003, unpaired two-tailed *t*-test. **c** Stereomicroscopic images of AAV6-Cre-GFP and AAV6-GFP treated scars at 14-dpi. The yellow dash lines indicate the scar edge. **d** Quantification of scar area based on histomorphometric analysis. Mean ± SD, *n* = 8, *p* = 0.0002, unpaired two-tailed *t*-test. **e** Masson’s trichrome stained vertical (upper panel) and transverse (lower panel) sections from AAV6-Cre-GFP and AAV6-GFP treated scars. The dash lines indicate scar width. **f** Quantification of scar width based on histomorphometric analysis. Mean ± SD, *n* = 5, *p* = 0.001, unpaired two-tailed *t*-test. **g** Fractal dimension and lacunarity analysis of AAV6-Cre-GFP and AAV6-GFP treated scars and adjacent normal skin. Mean ± SD, one-way ANOVA Tukey’s test, *n* = 8, *p* values from multiple comparisons are shown in the graph. Scale bars: **a**, **d** = 200 µm; **b** = 500 µm.

We injected AAV6 viral particles expressing Cre recombinase into the fascia surrounding wounds in N-cadherin floxed mice (*Ncad*^fl/fl^), which contains loxP sites flanking exon 1 of the *N-cadherin* gene. Thereafter, the transduced Cre-expressing fascia fibroblasts around wounds lost N-cadherin. As compared to the control virus, the Cre-expressing virus resulted in 65% reduction of N-cadherin expression in the scar region (Fig. 5a, b), and reduced scar area and width, evidenced by whole-mount macroscopic and histologic analyses (Fig. 5c–f). More importantly, transverse histologic views of scars clearly demonstrated that the centripetal pattern of collagen fibres around scar centres was disrupted by the N-cadherin patchy knockout (Fig. 5e lower panel). Fractal analysis measurements of control scars showed typical high fractal dimension (FD) and lower lacunarity (L) values in scars as compared to adjacent normal skin (Fig. 5g). N-cadherin knockout scars had a significantly more porous and less complex lattice (Fig. 5g), indicating that loss of N-cadherin improves wound quality.

To further confirm the essential role of N-cadherin in swarming that leads to scarring, we used an independent strategy to locally patchy knockout N-cadherin using CRISPR-Cas9. AAV6 viral particles expressing guide RNA targeting murine *N-cadherin* exon 1 or control virus were injected into the fascia surrounding wounds of full-body Cas9-expressing *R26*^Cas9^ mice. N-cadherin downregulation substantially reduced scar size in animals, as evidenced macroscopically and histologically (Supplementary Fig. 10). We further crossed *En1*^Cre^ mice with Cas9 knock-in mice to generate offspring in which only EPFs express Cas9. We locally knocked out N-cadherin in fascia EPFs by injecting viruses expressing specific guide RNA into the fascia surrounding wounds. Similar to our previous two experimental models, specific loss of N-cadherin in EPFs resulted in smaller scars and disrupted EPF swarming in animals (Supplementary Fig. 11).

**Fig. 6:**
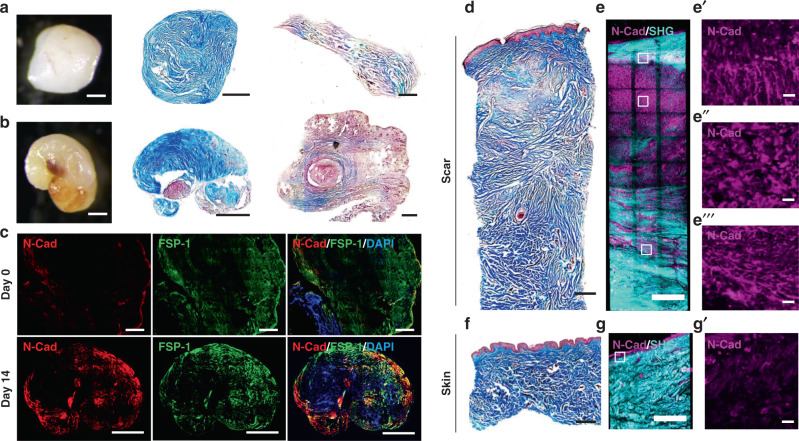
N-cadherin is involved in scar formation in human skin. **a**, **b** whole-mount bright-field image and Masson’s trichrome staining of SCADs made from human eyelid skin (**a**) or thigh skin (**b**) 14 days after culture (middle). The histology of respective fresh SCADs is shown in right column. *n* > 100. **c** Immunolabelling of N-cadherin (red) and FSP1 (green) of human SCAD at day 0 and day 14. **d**–**g** Masson’s trichrome staining and 3D immunostaining of N-cadherin on biopsy from human breast hypertrophic scar (**d, e**) or adjacent breast skin (**f, g**). N-cadherin (magenta), SHG (cyan). The enlarged images of the N-cadherin channel indicated by white boxes in **e**, **g** are shown in **e′**, **e″**, **e″′** and **g′**, respectively. Scale bars = 500 µm, except **e′**–**e″′**, **g′** = 20 µm.

To test if the N-Cadherin mechanism seen in SCADs and mice is clinically relevant, we generated SCADs from human skin-punch biopsies from eyelids or from thigh-wound margins. SCADs made from human tissues behaved in the same way as mouse. Human SCADs contracted and curved over 14 days, and ultimately stiffened with scars (Fig. 6a, b). As in mouse SCADs, N-cadherin expression within the contracted human SCADs overlapped with fibroblast aggregates (Fig. 6c).

We also examined the expression of N-cadherin in primary human hypertrophic breast scars (Fig. 6d) by whole-mount immunostaining. N-cadherin was highly expressed in scar fibroblasts directly beneath the epidermis, (Fig. 6e–e″, Supplementary Movie 12). N-cadherin-positive fibroblasts aligned in fibres in the more mature scar tissue in the deepest areas (Fig. 6e″′ and Supplementary Movie 12). Adjacent normal breast skin had healthy dermal patterns (Fig. 6f) and minimal N-cadherin expression (Fig. 6g, g′).

## Discussion

**Fig. 7:**
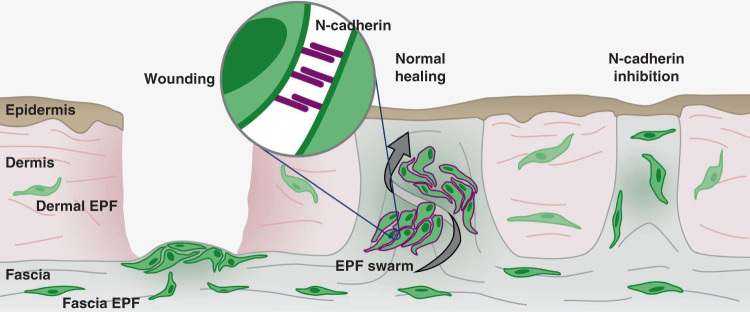
Scheme of N-cadherin mediated fascia EPF swarms upon wounding and scarring. upon deep wounding, fascia EPFs assemble into aggregates and migrate collectively towards wound centre and then swarm towards epidermis by N-cadherin (purple) mediated cell-cell adhesion. Fascia EPF swarming triggers fascia mobilisation into wounds, contracts skin and drives scar formation. EPF swarms are absent in the tissues without fascia (e.g. oral mucosa) or when N-cadherin is inhibited by chemicals (eg. Exherin) or genetic mutations. The absence of collective migration of fascia EPF swarms results in less scar formation.

We recently demonstrated that the key scar-forming cells reside deep in the subcutaneous fascia^9^ but their movements had not been visualised in real-time and the mechanism was not explained. Here we used in vivo and ex vivo live imaging to uncover their novel mode of movement and key molecular player and can thus propose a model of mammalian scarring involving N-cadherin expression and cell swarming towards formative scar centres (Fig. 7). Fascia EPFs upregulate N-cadherin upon wounding. The elevated intercellular adhesion is required for the aggregation and collective migration of fascia EPFs, and subsequent swarming towards wound centres. Swarming of fascia EPF triggers fascia mobilisation into wounds, contracts the skin and drives scar formation. In the tissues without fascia (eg. oral mucosa) or when N-cadherin is inhibited chemically (eg. Exherin) or genetically, fascia EPF swarming is absent, resulting in minimal scars.

The here proposed wound-induced mechanism helps to expound a series of earlier observations that indicate the provisional granulation tissue padding the wound bed is not responsible for contraction but rather that the active contraction comes from outside of the wound^18^. Indeed, excisions of the wound edge, which would disrupt swarming behaviours, relax skin contraction^19^.

N-cadherin has been shown to play a critical role in the invasion and migration of spheroid-myofibroblast aggregates into Collagen I gel or Matrigel in vitro^20^. Fibroblasts from Crohn’s disease strictures express enhanced levels of N-cadherin and N-cadherin overexpression has been shown to enhance fibroblast migration in vitro^21,22^. Previous studies also suggest wound-induced TGF-β1 signalling could be a trigger of elevated adherens junctions^20,21^. In addition, it has been demonstrated recently that upregulation of N-cadherin in glioma cells is dependent on p120-catenin. The adherens junctions stabilised by N-cadherin, β-catenin and p120-catenin appear to be crucial for the collective brain infiltration of glioma cells^23^. The exact molecular trigger of N-cadherin upregulation that drives fascia mobilisation requires further investigation.

The fascia system is not only present in skin. It is a continuous viscoelastic matrix that attaches, encloses, separates and interpenetrates all tissues and organs^24^. We therefore believe that fibroblast swarming is likely a general fibroblastic response to injuries in mammalian tissues and organs and not restricted to back-skin. Support to this tenet comes from functional experiments and in silico data from kidney and lung fibrosis. In these two models of fibrosis, Cadherin-11 is upregulated on fibroblasts in their connective tissues, and also serves as a translational biomarker for kidney fibrosis^25^. Cadherin upregulation in fibroblasts is directly linked to their invasion during lung fibrosis^26–28^. Fibroblast swarms within the injured internal organs may thus contribute to scarring in the same way as shown here in skin.

The recruitment of the fascia into wounds by fibroblast swarms has enormous clinical significance and opens a novel therapeutic space. Strategies that inhibit fascia fibroblast swarms from developing, for example by blocking the key molecule N-cadherin, could lead to therapeutics for preventing or reducing scar formation in human wounds.

## Methods

### Mice

C57BL/6J, *En1*^Cre^, *Wnt1*^Cre^, *ROSA26*^LSL-H2B-mCherry^ (*R26*^LSL-H2B-mCherry^)^29^ (JAX #023139), *ROSA26*^iDTR^ (*R26*^DTR^) (JAX #008040), N-cadherin floxed mice *Ncad*^fl/fl^ (JAX #007611, B6.129S6(SJL)-Cdh2^tm1Glr^/J) and Cas9 knock-in mice *R26*^LSL-Cas9-knockin^ (JAX #026175, B6J.129(B6N)-Gt(ROSA)26Sor^tm1(CAG-cas9*,-EGFP)Fezh^/J) strains were obtained from Jackson laboratories. The *ROSA26*^mTmG^ (*R26*^mTmG^) reporter mice were from Stanford University. *En1*^Cre^ or *Wnt1*^Cre^ transgenic mice were crossed with *R26*^mTmG^ or *R26*^LSL-H2B-mCherry^ reporter mice. Cas9-expressing Gt(ROSA)26Sor^tm1(Cas9)Rad^ (*R26*^Cas9^) mice were obtained from R. Rad (Institute of Molecular Oncology and Functional Genomics, Technical University of Munich, Germany). Animals were housed at the Helmholtz centre animal facility. The rooms were maintained at constant temperature and humidity with a 12-h light cycle. Animals were allowed food and water ad libitum. All animal experiments were reviewed and approved by the Government of Upper Bavaria and registered under the project ROB-55.2-2532.Vet_02-16-61 and ROB-55.2-2532.Vet_02-19-23, and conducted under strict governmental and international guidelines. This study is compliant with all relevant ethical regulations regarding animal research. Both male and female mice were used in the study. For wounding experiment with silicone splints in adult mice, the mice were wounded during telogen hair cycle stage.

### Human skin samples

Fresh human skin and scar biopsies, including 2 hypertrophic scars, from various anatomic locations, were collected from 71 donors between 18 and 65 years of age, through the Section of Plastic and Aesthetic Surgery, Red Cross Hospital Munich (reference number 2018–157), and by the Department of Dermatology and Allergology, Klinikum rechts der Isar, Technical University Munich (reference number 85/18S). Informed consent was obtained from all subjects prior to skin biopsies. Upon collection, these samples were directly processed for tissue culture or fixed with PFA and then processed for cryosection or paraffin section followed by histological or immunofluorescent analyses.

### Scar-like tissue in a dish assay

Full-thickness back-skin including fascia was collected from C57BL/6J or two-colour membrane reporter *En1*^Cre^*;R26*^mTmG^ or nuclear reporter *En1*^Cre^*;R26*^LSL-H2B-mCherry^ neonates at postnatal day 0–1, to allow a homogeneous fascia thickness. The harvested tissue was washed twice with cold DMEM/F-12 (Thermo Fisher Scientific 11320074) medium to remove contaminating blood, and then washed once with Hank’s Balanced Salt Solution (HBSS, Thermo Fisher Scientific 14175095). After careful removal of ventral non-skin tissue with a surgical scalpel, round skin pieces were cut out with a disposable Ø 2 mm biopsy punch (Stiefel 270130) down to bellow the panniculus carnosus muscle, and cultured in 200 µl of DMEM/F-12 medium containing 10% FBS, 1x GlutaMAX (Thermo Fisher Scientific 35050038), 1x Penicillin/streptomycin (Thermo Fisher Scientific 15140122), and 1x MEM non-essential amino acids (Thermo Fisher Scientific 11140035) in 96-well plates, in a humidified 37 °C, 5% CO_2_ incubator. (Note: the 2 mm skin pieces were cultured submerged in medium with dermal side face up, but not floating at the liquid-air interface). Fresh medium was supplied every other day and the skin tissues were harvested at the indicated time points (day 1–5 after culture), with the fresh tissues serving as day 0 control, and fixed in 2% PFA overnight at 4 °C. After washing in PBS, the tissues were embedded and frozen in optimal cutting temperature compound and 6 µm cryo-sections were prepared with a cryostat.

Oral SCADs were made from Ø 2-mm full-thickness cheek biopsies of neonates (postnatal day 0–1) of two-colour membrane reporter *Wnt1*^Cre^*;R26*^mTmG^ or nuclear reporter *Wnt1*^Cre^*;R26*^LSL-H2B-mCherry^ mice. Oral SCADs were cultured in a same way as the back-skin SCADs, with buccal mucosa face up.

Human SCADs were generated by incorporating the deep dermal and subcutaneous layers that contain fascia, but without epidermis, cultivated under same conditions as mouse SCADs. Tissues were harvested 14 days after culture, and processed for histology and immunofluorescence staining.

### SCAD chimera assay

SCAD biopsies were taken from neonatal back-skin of *En1*^Cre^*;R26*^mTmG^ mice with a Ø 4-mm biopsy punch. Thereafter, the 2-mm centre was excised from the 4-mm *En1*^Cre^*;R26*^mTmG^ SCAD with a Ø 2-mm biopsy punch, and replaced by a 2-mm SCAD generated from the Cre-negative littermates, in which the entire tissue is RFP labelled. Resultant chimeric SCADs were cultured in our standard conditions. The migratory behaviour of GFP^+^ EPFs into RFP^+^ inner SCAD was documented by live imaging. The possibility that the injury induced upregulation of factors that may promote migration cannot be ruled out.

### LC-MS/MS proteomic analysis

Scar tissues were harvested for proteomics from day 5 SCADs, and from in vivo 2-mm full-thickness excisional wounds on P2 neonates and at day-14 after wounding. After removing the epidermis, scar tissues were washed, pooled and homogenised. After centrifugation, proteins were extracted in Guanidinium Buffer (6 M Guanidinium chloride, 10 mM TCEP, 40 mM CAA, 100 mM Tris pH 8.5). Peptides from LysC and trypsin proteolysis (1:75), were purified on SDB-RPS material stage-tips and data was acquired on a Quadrupole/Orbitrap type Mass Spectrometer (Q-Exactive, Thermo Scientific)^30^. MS raw files were processed by MaxQuant^31^ (version 1.5.3.20) and MaxQuant output tables were analysed using the Perseus software suite^32^ (version 1.5.8.7). The identified proteins were filtered for Matrisome proteins^33^. Thereafter, network analysis was performed with Cytoscape software^34^ (version 3.6.1). All other statistical and bioinformatics operations, such as normalisation, heat-map generation, string-network mapping and multiple-hypothesis testing corrections, were performed with the Perseus software suite. The mass spectrometry proteomics data have been deposited to the ProteomeXchange Consortium via the PRIDE partner repository with the dataset identifier PXD016068.

### Local N-cadherin knockout in *R26*^Cas9^ or *En1*^Cre^;*R26*^Cas9-knockin^ or *Ncad*^fl/fl^ wounds

A guide RNA (gRNA) targeting exon 1 of mouse N-cadherin was designed with the Benchling tool. The adeno associated virus serotype 6 (AAV6) expressing gRNA targeting murine N-cadherin (TCCGGCACATGGAGGCGGAG) was created by cloning gRNA into the SAP1 sites of pAAV-U6-sgRNA-CMV-GFP (Addgene #85451^35^). N-cadherin gRNA expressing AAV6 (AAV6-NcadgRNA-GFP) was produced by transfecting the AAVpro 293T Cell Line (Takara Bio 632273) with pAAV-U6-NcadgRNA-CMV-GFP, pRC6 and pHelper plasmids from the AAVpro Helper Free System (Takara Bio 6651). Cre-expressing AAV6 (AAV6-Cre-GFP) was purchased from Addgene (Addgene #68544^36^). Transfection was performed with PEI transfection reagent and viruses were harvested 72 h post-transfection. AAV6-NcadgRNA-GFP or AAV6-Cre-GFP or control AAV6-GFP viruses were extracted and purified with an AAVpro purification kit all serotypes (Takara Bio 6666).

Two Ø 2-mm full-thickness excisional wounds were created on the back of anaesthetised 13-day-old full-body Cas9-expressing *R26*^Cas9^ mice or 5 days old *En1*^Cre^*;R26*^**Cas9-knockin**^ mice that express Cas9 in EPFs. CRISPR/Cas9-based local N-cadherin ablation was performed by subcutaneously injecting 20 µl of AAV6-gRNA-GFP virus at a viral titre of 1 × 10^12^/ml at the area between the two wounds (~5 mm in distance) at day 0, 5 and 10 post-wounding. The small bleb (fluid pocket) resulted from the injection reached the edges of both wounds. The mice received subcutaneous injection of the AAV6-GFP virus without gRNA as control.

Similarly, two Ø 2-mm full-thickness excisional wounds were created on the back of anaesthetised 5 days old homozygous N-cadherin floxed *Ncad*^fl/fl^ mice containing loxP sites flanking exon 1 of the *N-cadherin* gene^37^. Local N-cadherin ablation was performed by subcutaneously injecting 20 µl of AAV6-Cre-GFP virus at a viral titre of 2 × 10^12^/ml at the area between the two wounds at day 0, 5 and 10 post-wounding. The fluid pocket after injection reached the edges of both wounds. The mice received subcutaneous injection of the AAV6-GFP virus as control.

Scar tissues were harvested at day 14 post-wounding. Scar images were documented with a Leica M50 stereo-microscope (Leica) at ×4 magnification. Subsequently, the scar tissue was processed for cryo-sections for histology and immunofluorescence.

### Antibodies and reagent

The following antibodies were used: N-cadherin (clone GC4, Sigma-Aldrich C3865, 1:100 dilution), FSP1 (Abcam ab58597, 1:100 dilution), PDGFRα (R&D Systems AF1062, 1:50 dilution), Collagen I (Rockland 600–401–103–0.1, 1:150 dilution), Collagen III (Abcam ab7778, 1:150 dilution), Fibronectin (Abcam ab23750, 1:200 dilution), α-SMA (Abcam ab5694, 1:100 dilution), CD31 (Novus Biologicals NB100-2284, 1:500 dilution), EpCAM (Abcam ab92382, 1:500 dilution), Lyve-1 (Abcam ab14917, 1:500 dilution), Myf-5 (clone C-20, Santa Cruz sc-302, 1:500 dilution), CD45 (clone IBL-3/16, Abcam ab23910, 1:500 dilution), FABP4 (clone EPR3579, Abcam ab92501, 1:500 dilution), F4/80 (Abcam ab90247, 1:500 dilution), Cytokeratin 14 (clone EPR17350, Abcam ab181595, 1:200 dilution), Integrin α_v_ (clone EPR16800, Abcam ab179475, 1:500 dilution), α1-catenin (clone EP1793Y, Abcam ab51032, 1:500 dilution), decorin (Abcam ab175404, 1:500 dilution), DLK-1 (Abcam ab21682, 1:200 dilution) and Ki67 (clone SP6, Abcam ab16667, 1:500 dilution). Fluorophore-conjugated secondary antibodies were purchased from Thermo Fisher Scientific (1:500 dilution). Exherin (ADH-1) was from TargetMol (T2637).

### Masson’s trichrome staining

Cryo-sections were fixed in cold acetone (−20 °C) for 5 min and incubated overnight in Bouin’s solution (Sigma-Aldrich HT10132) at room temperature. After washing in cold tap water, the sections were stained with a working concentration of Weigert’s Iron Hematoxylin (Sigma-Aldrich HT1079) for 5 min. Thereafter, the sections were treated with a Masson’s trichrome stain kit (Sigma-Aldrich HT15) by sequentially incubating at room temperature in Biebrich scarlet-acid fuchsin solution for 5 min, working concentration of Phosphotungstic /Phosphomolybdic acid for 5 min, aniline blue solution for 10 min, and 1% acetic acid for 2 min. After dehydration, the sections were cleared with Roti-Histol and mounted with a Roti-Histokitt (Roth 6640 and 6638 respectively).

Cell counting was performed with ImageJ. Briefly, trichrome images were converted to CMYK and the black channel was used to count the iron haematoxylin stained nuclei. Regions of interest covering the scar area were delimited and processed using the functions of subtract background (rolling ball = 50 px), enhance contrast (saturated = 0.1 normalised), Unsharp Mask (Radius = 2, Mask = 0.6), Median (radius = 1) and the auto-threshold method Minimum. Watershed function was used in the binary images and the particles were counted (size threshold = 30–200 px^2^).

For the quantification of scar areas, trichrome images were converted to CMYK and the cyan channel was used to analyse the blue-stained collagens.

### Immunofluorescence staining

Cryo-sections were fixed in cold acetone for 5 min and blocked with 5% BSA in PBS for 1 h. Thereafter, sections were incubated overnight with primary antibodies at 4 °C. After three PBS washes, sections were incubated with appropriate fluorophore-conjugated secondary antibodies (Thermo Fisher Scientific) at room temperature for 1 h. For double staining, sections were incubated at room temperature with the second primary antibody for 2 h, and appropriate secondary antibody for 1 h. Nuclei were counterstained with DAPI for 3 min. Coverslips were mounted with Fluoromount-G (Thermo Fisher Scientific 00–4958–02). Photomicrographs were taken with a Zeiss AxioImager microscope with AxioVision software (Carl Zeiss), or a Zeiss laser scanning microscope LSM710 with Zen software (Carl Zeiss).

### Whole-mount bright-field imaging

SCAD tissues were fixed with 2% PFA at 4 °C overnight, and washed three times with PBS. Whole-mount bright-field images were taken with a Leica M50 stereo-microscope (Leica) with a Leica DFC310 FX camera (Leica) and saved with Leica Application Suite (v4.8).

### 3D imaging

(1) By laser scanning microscopy with tissue-clearing: whole-mount samples were stained and cleared by a modified 3DISCO protocol. In short, fixed samples were pre-incubated in Dulbecco’s Phosphate-Buffered Saline (DPBS, Thermo Fisher Scientific 14190169) containing 0.2% gelatin (Sigma G1393), 0.5% Triton-X100 (Sigma X100) and 0.01% Thimerosal (Sigma T8784) (PBS-GT), for 24 h at room temperature. Antibodies: anti-elastin (Abcam ab21610), anti-fibronectin (Abcam ab23750) and anti-collagen type I (Rockland 600–401–103), were pre-labelled with Alexa Fluor 488, 594 and 647 dyes (Thermo Fisher Scientific A20181, A20184, A20186) according to the manufacturer’s instructions. The samples were incubated, rotating, with the labelled antibodies in PBS-GT (1:1000) for 24 h at room temperature. After washing in PBS-GT, samples were first dehydrated in an ascending THF (Sigma 186562) series (50%, 70%, 80%, 3 × 100%; 30 min each), then cleared in dichloromethane (Sigma 270997) for 30 min and eventually immersed in benzyl ether (Sigma 108014). Cleared samples were imaged in 35 mm glass-bottom dishes (ibidi 81218) under a laser scanning confocal microscope (Zeiss LSM710, Carl Zeiss, Germany). Raw data was processed and optimised for visualisation, adjusting brightness and contrast with Imaris software (v9.1.0, Bitplane, UK).

(2) By multi-photon microscopy without tissue-clearing: whole-mount samples were immuno-labelled and embedded in 2% NuSieve GTG agarose (Lonza 859081) in a 35 mm dish (Falcon 351008) and were imaged under a Leica SP8 MP (Leica, Germany). Tiles were merged offline using the Leica Application Suite X (v4.8, Leica) with smooth overlap blending, and data were visualised with Imaris software (v9.1.0, Bitplane, UK) using contrast and brightness adjustments.

### Live imaging using multi-photon microscopy

Full-thickness skin pieces were collected as described above and imaged with a Leica SP8 MP (Leica, Germany). In brief, samples were embedded in 4% agarose (Lonza, 859081) in a 35-m dish (Corning, 351008) and submerged in imaging medium (phenol-red-free DMEM/F-12 (Thermo Fisher Scientific, 21041025) containing 10% KnockOut Serum Replacement (Thermo Fisher Scientific, A3181501), 1x GlutaMAX (Thermo Fisher Scientific, 35050038), 1x Penicillin/streptomycin (Thermo Fisher Scientific, 15140122), and 1x MEM non-essential amino acids (Thermo Fisher Scientific, 11140035)). A 25x water-dipping objective (HC IRAPO L 25x/1.00 W) was used in combination with a tunable laser (Spectra Physics, InSight DS + Single). Second harmonic signal was collected with external hybrid photodetectors with a HC 405/150 filter. Green, orange, red, and far-red signals were collected using a 525/50, 585/40, 624/40, and 650/50 bandpass filters.

A modified incubation system, with heating and gas control (ibidi 10915 and 11922), was used to guarantee physiologic and stable conditions during imaging (35 °C, 5% CO_2_). Z-stacks were recorded in 15 min intervals.

### Intravital multi-photon imaging

Dorsal skinfold chamber was implanted as described before^38^. In brief, mice were anesthetised and the dorsal back skin was shaved, depilated and disinfected. A fold of the dorsal skin was examined for major vessels and relocated to spare them during preparation of the wound. The sterilised skinfold chamber (APJ Trading Co, Inc; Ventura, CA) was sutured to the skin on the upper side using 4/0 braided sutures (Chirlac #PG 0203, Vitrex Medical, Denmark) and fixed with the three bolts and nuts. A full-thickness wound was created in the middle of the observation window using a 3-mm biopsy punch (Stiefel) carefully removing epidermis, dermis and panniculus carnosus layer. After flushing with physiologic saline, a sterile coverslip was used to cover the wound.

The wound was imaged 14 days post wounding for a period of 6 h using a Leica SP8 MP (Leica, Germany) equipped with a 25x water-dipping objective (HC IRAPO L 25x/1.00 W) and a tuneable laser (Spectra Physics, InSight DS + Single). Fluorescent signal was generated at 960 nm (SHG, GFP) and 1050 nm (tdTomato) and collected with external hybrid photodetectors using the following bandpass filters: SHG: 405/150; GFP: 525/50; tdTomato: 585/40.

Mice were anesthetised with 1–3% Isoflurane and maintained for the time of intravital imaging. A subcutaneous depot of 200 µL physiologic saline was injected to prevent dehydration. Mice were placed in lateral position on a heated plate and vital parameters (ECG, respiration, rectal temperature) were monitored continuously (75–1500, Harvard Apparatus, MA). The dorsal skinfold chamber was fixed on a custom made metal bracket, which was stably mounted to the stage of the microscope. A thin circular barrier of silicon grease (Korasilon, Kurt Obermeier, Germany) was applied to the coverslip to keep the immersion water from draining.

### Orientation vector field analysis

Vector fields were generated using ImageJ plugin OrientationJ 2.0.4 – Vector Field^39^. In brief, frame images of the green channel (EPFs) from intravital microscopic recording were subjected to maximum intensity projection. Substacks were created with every 10 frames of the recording. Orientation vector fields were applied with vector field grid size of 150 pixels with 100% length of scale vector. Resulting vector trajectories were converted into masks and overlaid on the original frame image.

### Contour line trajectory analysis

Contour lines were derived using ImageJ plugin Interactive 3D surface plot. Maximum intensity substacks were computed using the frame images of the green channel (EPFs) corresponding to 0, 3 and 6 h of the intravital microscopic recording. These frames were subjected to 3D projection with full transparency to create a pseudo z stack. To mark the boundaries of the pseudo z stack, contour lines corresponding to leading front of the frames were overlaid and 3D surface plot were rendered. Contour lines were generated with following parameters: Grid size - full size of the image, Smoothing - 130, Lighting - 0.50 and perspective - 0.0. Resulting contour lines were displayed using standard fire lookup tables from LUT library. The gradient lines perpendicular to the contour lines are used to predict the trajectory of migrating EPF swarms.

### Automated and manual cell tracking

Automated cell tracking was performed on SCADs made from nuclear reporter lines (*En1*^Cre^*;R26*^LSL-H2B-mCherry^ or *Wnt1*^Cre^*;R26*^LSL-H2B-mCherry^) using Imaris software package (v9.1.0, Bitplane, UK). Tracks were generated from 4D data using the fluorescence intensity-based surface detection tool. The resulting data that represents the nuclei of Cre-positive cells were filtered for good quality. Tracks were visualised in a time-coded colour representation, ranging from purple to red. Only the last 12 time points of the respective tracks are shown for better visibility and to prevent overcrowded images and videos.

Manual cell tracking was performed on SCADs from two-colour membrane reporter lines (*En1*^Cre^*;R26*^mTmG^ or *Wnt1*^Cre^*;R26*^mTmG^) with ImageJ using the Manual Tracking plugin (version 2.1.1), since the cell boundaries in the membrane reporter system can’t be accurately segmented and followed by the automatic tracking software. In brief, the channels of 3D-Time lapse datasets were split and subjected to maximum intensity projection. Migration of individual cells was tracked over time. Trajectories and individual track information with coordinates were exported as TIFF and excel file respectively. Graphical visualisation and analysis of these trajectories were performed using R. Iterative loops to generate a colour-ramp for each track as a function of time and embedded into respective coordinates (Blue = first time point; Red= last time point).

### Fractal analysis of images

We use fractal analysis to quantify the complexity of cellular arrangement and extracellular matrix. In this analysis, the fractal dimensions and lacunarity values measure the complexity and porosity of shapes respectively. Complex arrangements score higher fractal dimension values than simpler arrangements, while porous structures score higher lacunarity values than smoother ones.

Images were analysed with ImageJ (ImageJ v1.47) to produce values that were analysed using GraphPad 6. For fractal analysis of collagens, trichrome images were converted to CMYK and the cyan channel was used to analyse the blue-stained collagens. Images were processed using the subtract background (rolling ball = 50 px), enhance contrast (saturated = 0.1 normalised), Unsharp Mask (Radius = 2, Mask = 0.6), Median (radius = 1) and the auto-threshold method Default functions. Fractal dimension and lacunarity values were calculated using the FracLac Plugin with a pixel threshold of 0.40.

### Particle image velocimetry analysis of images

Particle image velocimetry (PIV) has been used to describe collective cell migration^40^. Using the ImageJ plugin^41^ we performed PIV on the GFP/RFP signal from live-imaging video stacks of *En1*^Cre^*;R26*^mTmG^ dorsal SCAD or *Wnt1*^Cre^*;R26*^mTmG^ oral SCADs. Successive video frames of 30 min intervals from 24 h videos were subjected to iterative (cross-correlation) PIV on 64 × 64, 32 × 32 and 16 × 16 pixel sub-windows. Normalised median test (noise = 0.2, threshold = 5) post-processing was used to decrease the background noise. Calculated magnitude vectors were then used to measure direction and velocity.

### Enrichment index, displacement, velocity and similarity measures of images

EPF enrichment index was calculated by mean fluorescence intensity (MFI) of GFP channel (EPF) divide by the sum of MFI of GFP channel and MFI of RFP channel (ENF). Similarly, WPF enrichment index in oral mucosa SCAD was calculated by MFI of GFP channel (WPF) divide by the sum of MFI of GFP channel and MFI of RFP channel (WNF). Displacement measures were based on five landmarks between alternate frames from live imaging. Mean displacement values were added up and plotted versus time. Using the tracked trajectories, we calculated the distance between positions of the same cell at consecutive time points in 3D. To measure movement similarity of neighbouring cells we first determined the movement vector for every cell between consecutive time points. Then we compared this vector to all movement vectors of neighbouring cells by calculating the angles between these vector pairs in 3D. Finally, we averaged all angles to produce one movement-similarity score. To identify neighbours of individual cells we applied a Delaunay triangulation on 3D cell coordinates^42^.

### Statistics and reproducibility

GraphPad Prism was used for all statistical analyses except proteomics. Unless otherwise indicated, mean ± SD values are reported in the graphs. The exact statistical analyses used to quantify data, the exact values of *n*, and the exact *p* values are stated in the respective figure legends. For simplicity, *p* values below 0.0001 were stated as equal to 0.0001. All experiments were performed at least three times independently with similar results.

### Reporting summary

Further information on research design is available in the Nature Research Reporting Summary linked to this article.

## Supplementary information

Supplementary Information

Description of Additional Supplementary Files

Supplementary Movie 1

Supplementary Movie 2

Supplementary Movie 3

Supplementary Movie 4

Supplementary Movie 5

Supplementary Movie 6

Supplementary Movie 7

Supplementary Movie 8

Supplementary Movie 9

Supplementary Movie 10

Supplementary Movie 11

Supplementary Movie 12

Supplementary Data 1

Reporting Summary

## Data Availability

The proteomics data have been deposited in the ProteomeXchange Consortium via the PRIDE partner repository with the dataset identifier: PXD016068. The data that support the findings of this study are available from the authors on reasonable request.
