# Supplementary material for: Injury triggers fascia fibroblast collective cell migration to drive scar formation through N-cadherin
Source: Nat Commun. 2020 Nov 6;11:5653. doi: 10.1038/s41467-020-19425-1 (PMC7648088; doi:10.1038/s41467-020-19425-1)
Supplement: Supplementary file 1 — Supplementary Information [file 41467_2020_19425_MOESM1_ESM.pdf]

Injury triggers fascia fibroblast collective cell migration to drive scar formation through N-cadherin

Dongsheng Jiang[1,ξ], Simon Christ[1,ξ], Donovan Correa-Gallegos[1,ξ], Pushkar Ramesh[1,ξ], Shruthi Kalgudde Gopal[1], Juliane Wannemacher[1], Christoph H. Mayr[2], Valerio Lupperger[3], Qing Yu[1], Haifeng Ye[1], Martin Mück-Häusl[1], Vijayanand Rajendran[1], Li Wan[1], Juan Liu[1], Ursula Mirastschijski[4,5], Thomas Volz[6], Carsten Marr[3], Herbert B. Schiller[2,7], Yuval Rinkevich[1,7,*]

SUPPLEMENTARY INFORMATION

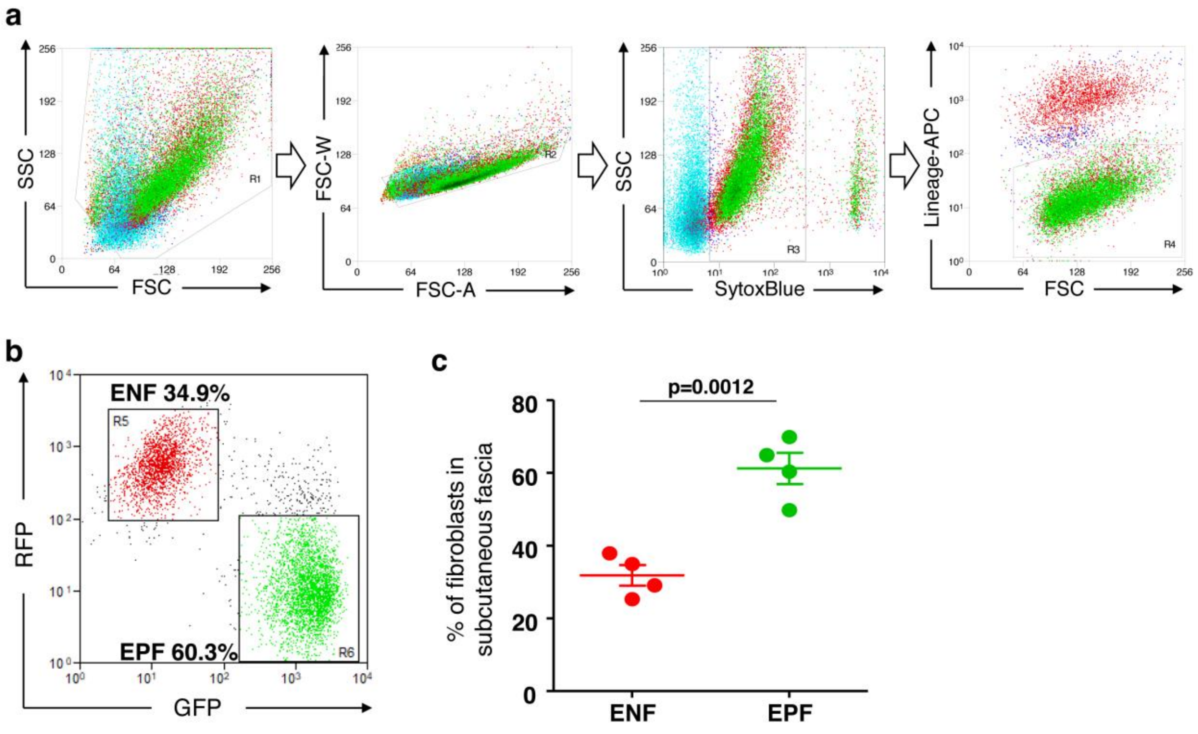

Supplementary Figure 1. EPF and ENF composition in subcutaneous fascia.

a, Subcutaneous fascia was separated from the back-skin of *En1*^{Cre}*;R26*^{mTmG} neonates (P0-P1). Single cell suspension was prepared from the pooled fascia and subjected to flow cytometric analysis. Sytox Blue was used to exclude dead cells, and APC-conjugated lineage markers (CD45, CD31, Ter119, EpCAM, Tie-2, Lyve-1) were used to exclude non-fibroblastic cells. **b**, in living Lin$^-$ population, GFP$^+$RFP$^-$ cells were gated as EPFs, and GFP$^-$RFP$^+$ cells were gated as ENFs. **c**, percentages of ENFs and EPFs in subcutaneous fascia. Mean ± SD, unpaired two-tailed *t*-test, p=0.0012, n=4.

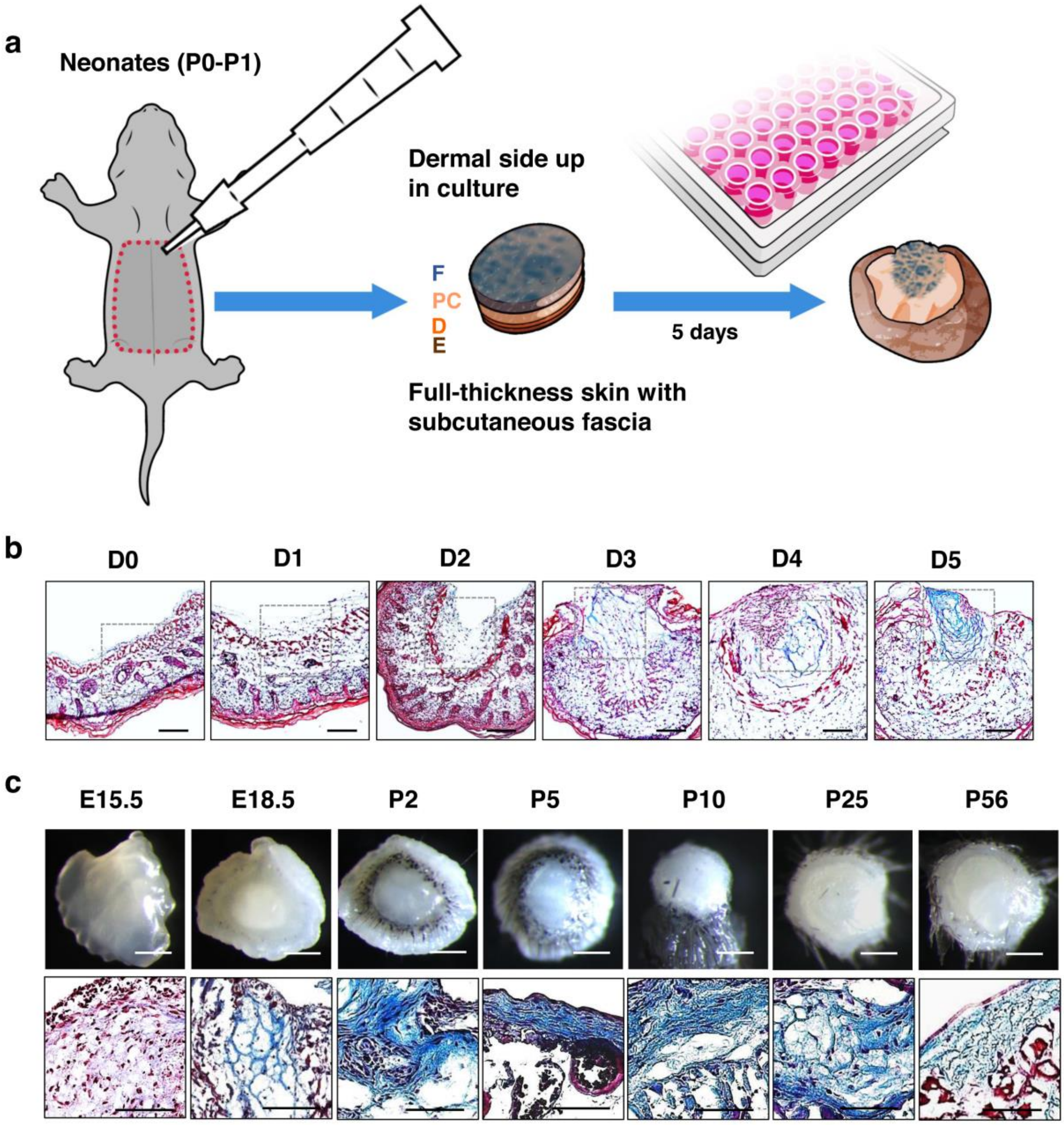

Supplementary Figure 2. SCAD model.

a, Key elements of the SCAD assay include (1) back-skin from new-born mice (postnatal day 0-1) for uniform scar formation, (2) full-thickness skin biopsy including subcutaneous fascia, (3) culturing with fascia side up. E, epidermis; D, dermis; PC, panniculus carnosus; F, subcutaneous fascia. **b**, Masson's trichrome staining of cryosections of SCADs from day 0 to day 5 of culture. Scale bars = 200 µm. **c**, SCADs from mice at various development stages. Whole-mount images (upper panel, scale bars = 500 µm) and Masson's trichrome staining (lower panel, scale bars = 200 µm) of SCADs using back-skin from embryos (E15.5, E18.5), new-borns (P2), juveniles (P5, P10, P25) to adults (P56).

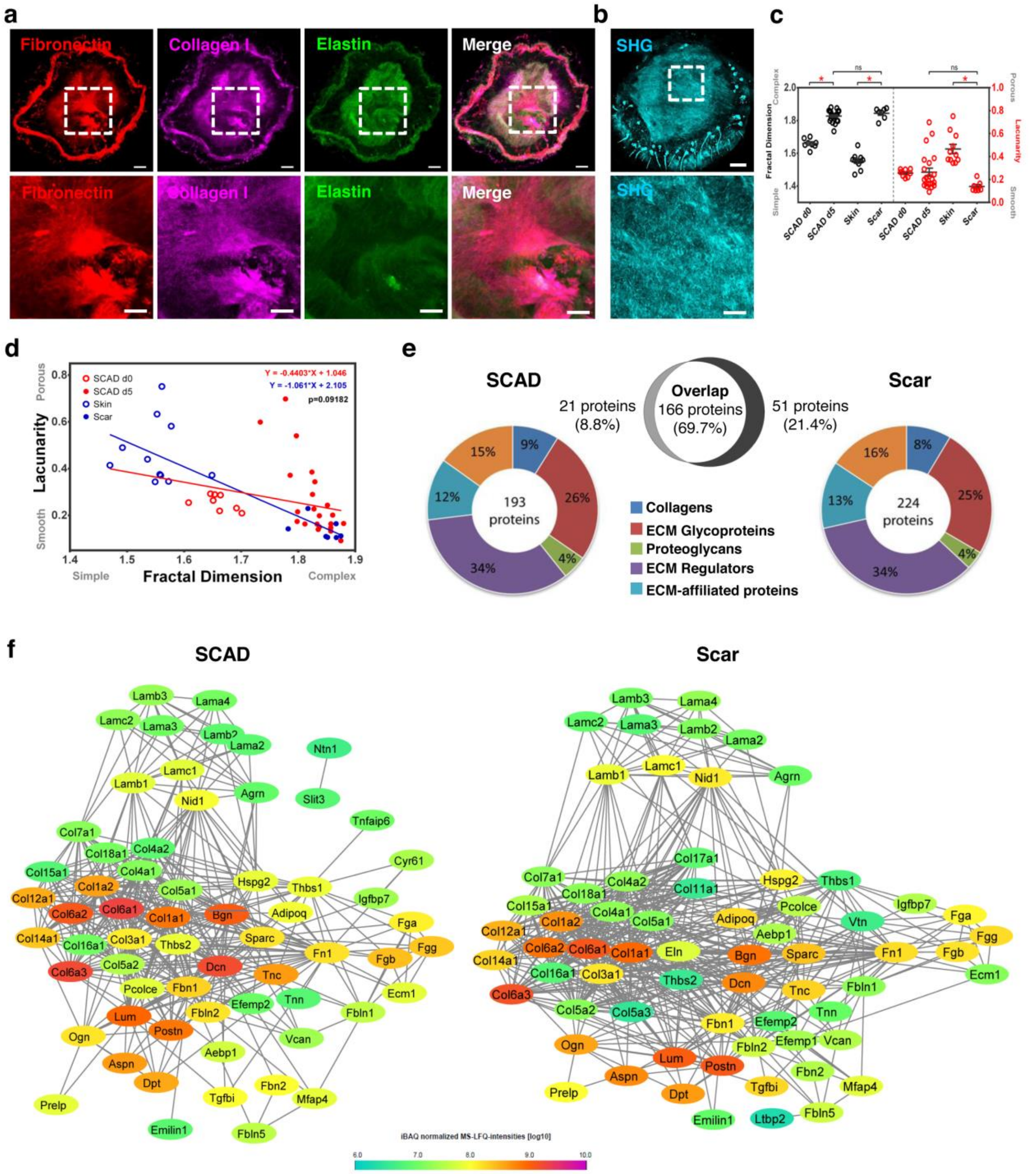

Supplementary Figure 3. SCAD recapitulates characteristics of *in vivo* fascia scar.
a, 3D immunostaining of fibronectin (red), collagen I (magenta) and elastin (green) in SCADs at low (upper panel) and high (lower panel) magnification. **b**, fibrillary collagens represented by second harmonic generation (SHG, cyan) signal in SCADs at low (upper) and low (lower) magnification. **c**, fractal dimension (left) and lacunarity (right) of day0 SCAD, day5 SCAD, *in vivo* skin, and *in vivo* scars. Mean ± SD, n=7/22/11/7, One-way ANOVA Tukey's test. *, p=0.0001; ns, not significant. **d**, regression lines showing similar changes in fractal values between SCAD and in vivo scars. **e**, 4438 proteins were identified by proteomic analysis. When filtered for Matrisome proteins, 193 proteins in SCADs and 224 proteins in *in vivo* scars remained, respectively. The pie chart shows the similar percentages of Matrisome proteins in the indicated categories and the Venn diagram shows the overlapping Matrisome proteins of SCAD and in vivo scars. **f**, a protein-protein interaction network showing the similarity of matrix proteins between SCAD and in vivo scars. The colour coding indicates the abundance of proteins. Scale bars: a, b upper panel = 100 µm, lower panel = 30 µm.

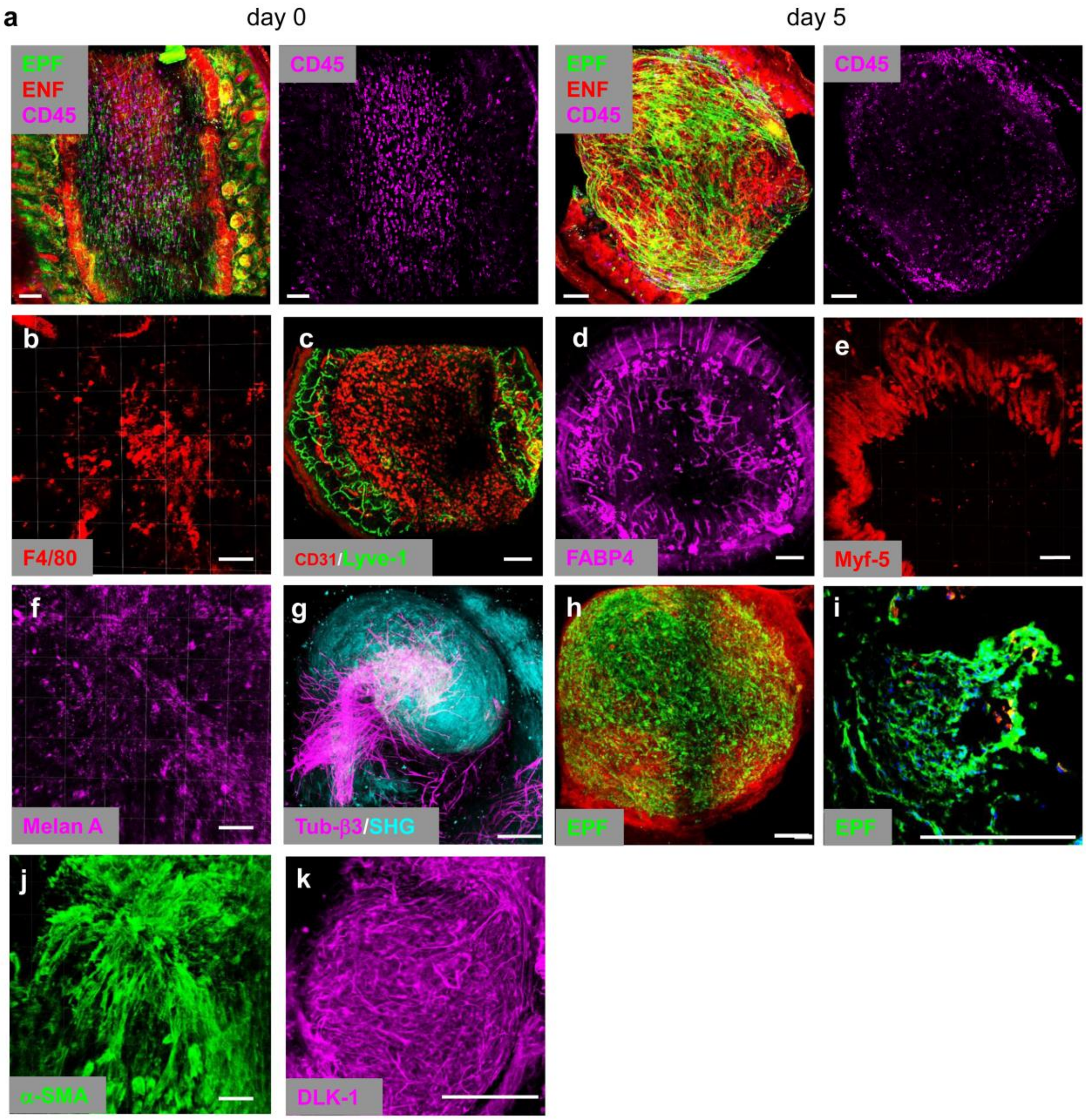

Supplementary Figure 4. Cellular component of SCAD.
a, 3D immunolabeling of CD45 (magenta) in day0 and day5 SCADs. **b-g**, 3D immunolabeling of F4/80 (**b**), CD31 (red) and Lyve-1 (green) (**c**), FABP4 (**d**), Myf-5 (**e**), Melan A (**f**), and Tubulin β3 (magenta) and SHG (cyan) (**g**) in SCADs. **h, i**, 3D (**h**) and 2D (**i**) fluorescence images of *En1*^{Cre}*;R26*^{mTmG} SCAD, EPFs in green. **j, k**, 3D immunolabeling of α-SMA (**j**) and DLK-1 (**k**) in SCAD. Images are representative of at least three biological replicates. Scale bars = 100 μm.

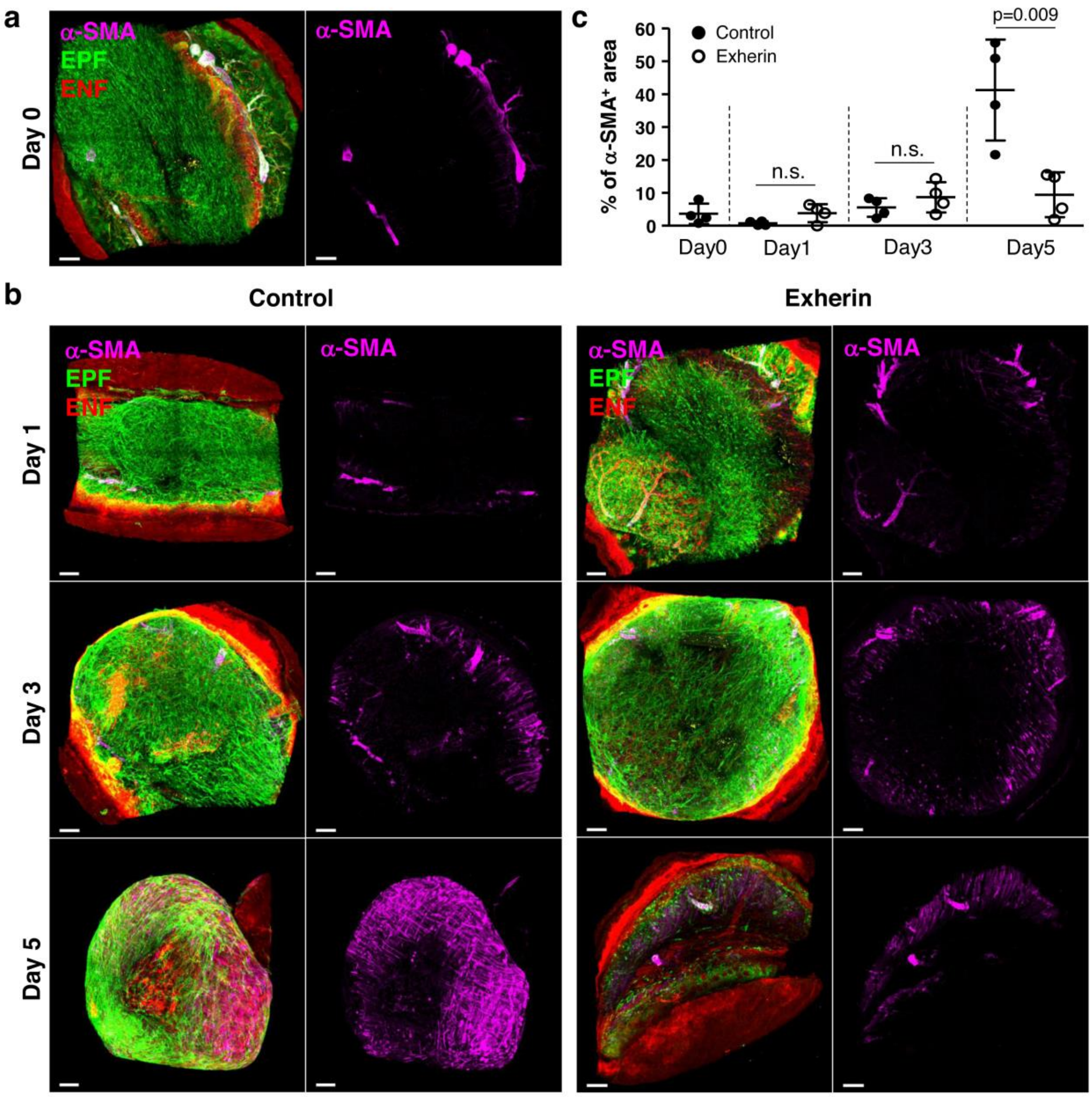

Supplementary Figure 5. α-SMA expression in SCAD.

3D immunolabeling of α-SMA in *En1*^{Cre},*R26*^{mTmG} SCAD before culture (**a**) or cultured 1, 3, 5 days with or without 500 µg/ml Exherin treatment (**b**). α-SMA in magenta, EPFs in green, and ENFs in red. **c**, quantitative analysis of percentage of α-SMA^{+} area in respective SCAD. Mean ± SD, unpaired two-tailed *t*-test, p=0.009, n = 4. Scale bars: 100 µm.

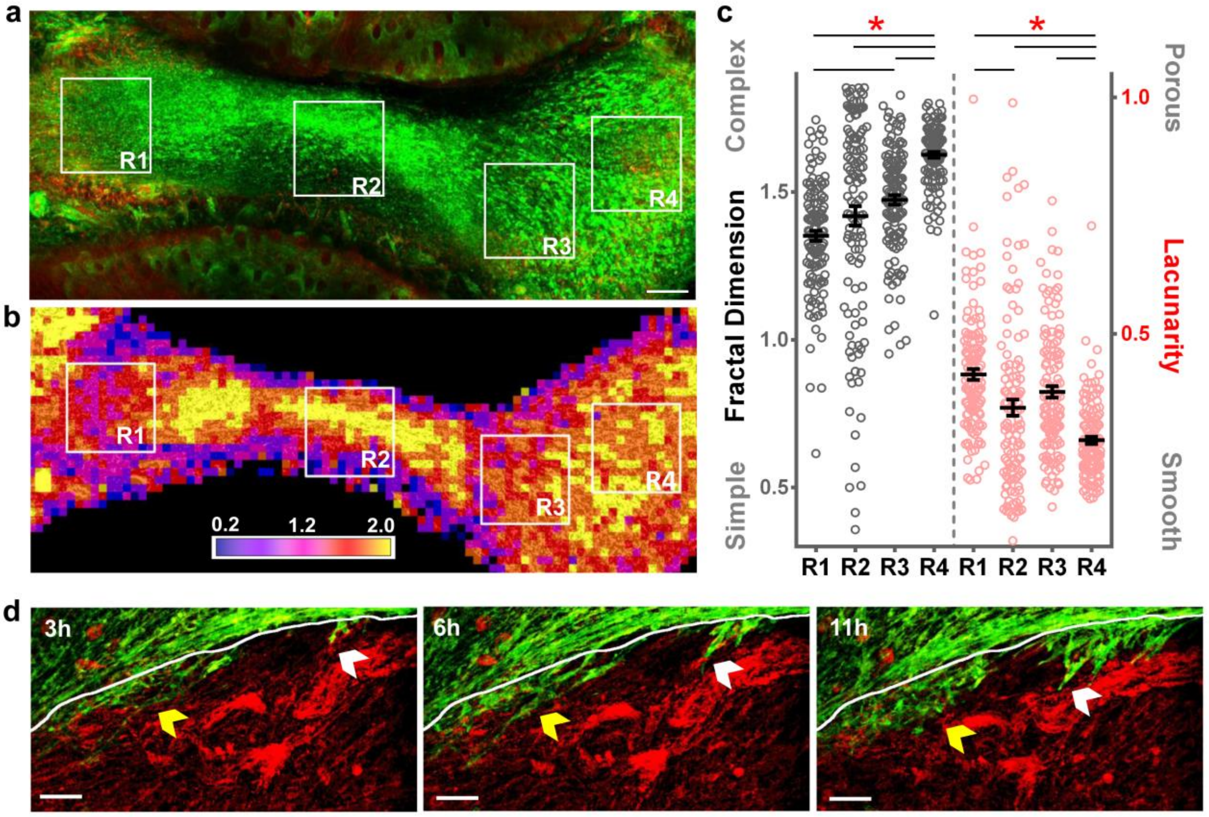

Supplementary Figure 6. Fascia EPFs aggregate in groups and migrate collectively at the onset of scarring.

a, snapshot of live imaging (0-24 h) of *En1*^{Cre}*;R26*^{mTmG} SCAD at 24 h in culture. **b**, fractal analysis of EPFs (GFP channel). Colour code indicates fractal dimension (FD) values from low (dark) to high (bright). **c**, FD (left) and lacunarity (right) values of regions R1-R4. Mean ± SEM of all subsampled values in each region. One-way ANOVA, Tukey's multiple comparisons test, *p=0.001, n=121 samples. **d**, snapshots (3h, 6h, 11h) of live imaging of a chimeric SCAD showing the migration of fascia EPF aggregates (Supplementary Movie 3). White lines indicate the border between the outer *En1*^{Cre}*;R26*^{mTmG} SCAD and inner grafted RFP only SCAD. The arrows indicate the invasion of fascia EPF aggregates into the inner region. The yellow and white arrows indicate two independent EPF aggregates. Scale bars: a = 100 µm; d = 50 µm.

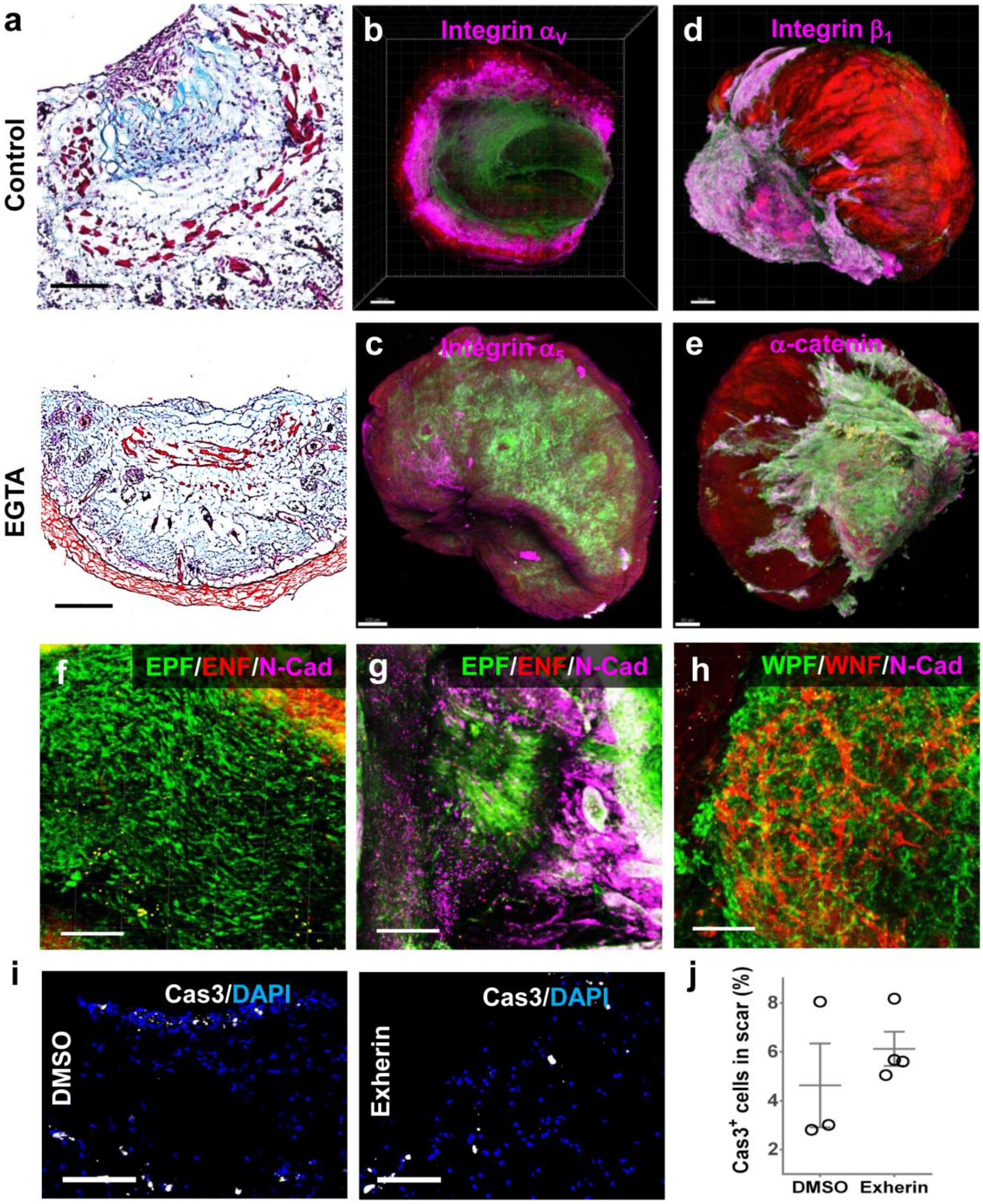

Supplementary Figure 7. N-cadherin mediated cellular adhesion of EPFs is essential for scar formation.

a, Masson's trichrome staining of control and 0.5 mM EGTA treated SCAD. **b-e**, 3D immunolabeling of integrin α_V (**b**), integrin α_5 (**c**), integrin β_1 (**d**), and α-catenin (**e**) in *En1*^{Cre}*;R26*^{mTmG} SCAD. **f-h**, 3D immunostaining of N-cadherin (magenta) in day0 (**f**) and day2 (**g**) *En1*^{Cre}*;R26*^{mTmG} dorsal SCAD, and day4 *Wnt1*^{Cre}*;R26*^{mTmG} oral SCAD (**h**). **i**, immunostaining of active caspase-3 in scar area of DMSO or 500 µg/ml Exherin treated SCAD. Cell nuclei were stained with DAPI. **j**, percentages of active caspase-3^{+} cells in scar area of DMSO or Exherin treated SCAD. n=3/4, p=0.4066, unpaired two-tailed *t*-test. Scales: a = 200 µm; b = 150 µm; c, f-i = 100 µm; d = 70 µm; e = 80 µm.

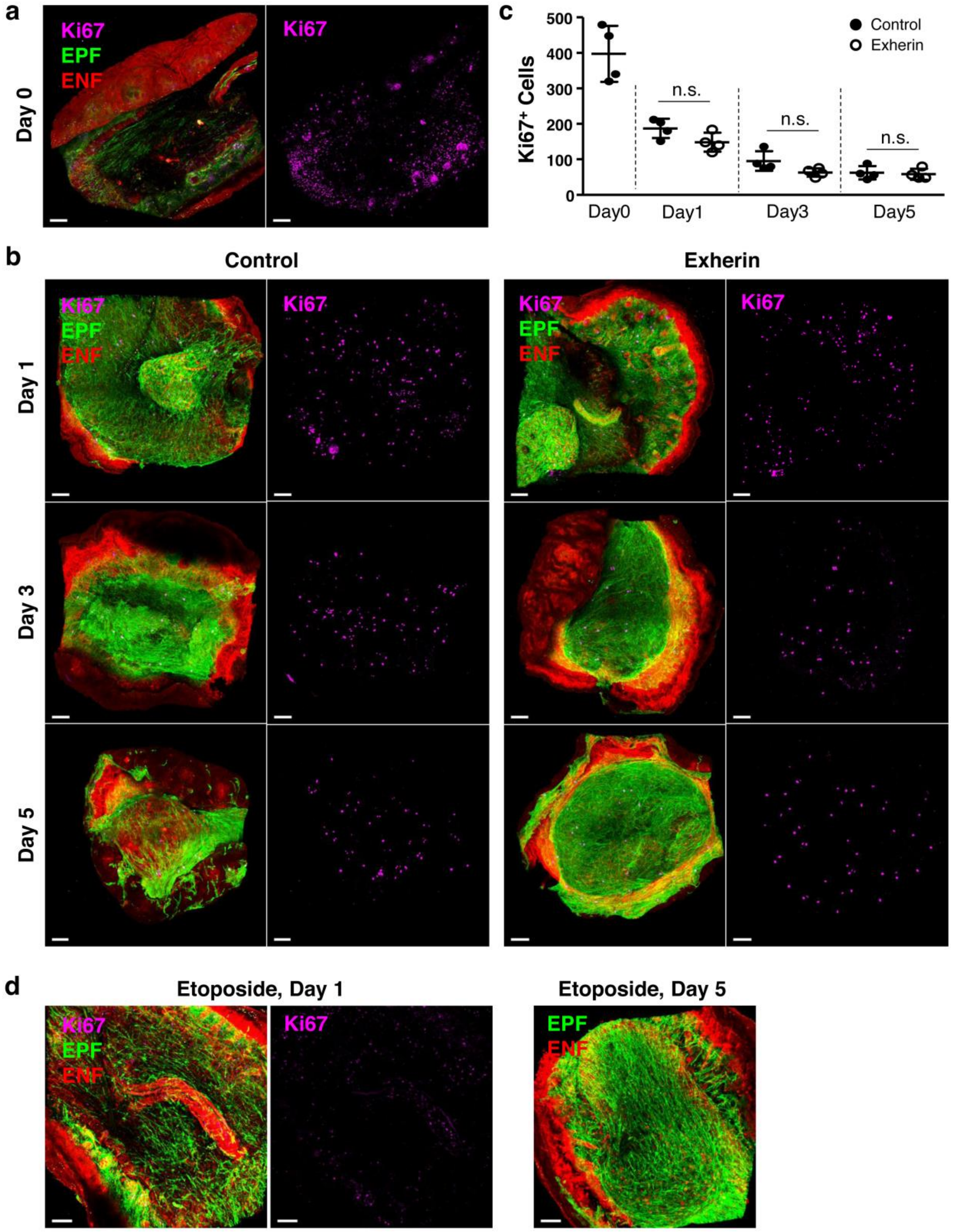

Supplementary Figure 8. Ki67 expression in SCAD.
3D immunolabeling of Ki67 in *En1*^{Cre},*R26*^{mTmG} SCAD before culture (**a**) or cultured 1, 3, 5 days with or without 500 µg/ml Exherin treatment (**b**). Ki67 in magenta, EPFs in green, and ENFs in red. **c**, quantitative analysis of number of Ki67^{+} cells in respective SCAD. Mean ± SD, unpaired two-tailed *t*-test, n = 4; n.s., not significant. **d**, SCAD treated with 100 µM etoposide at day 1 and 5 after culture. Scale bars: 100 µm.

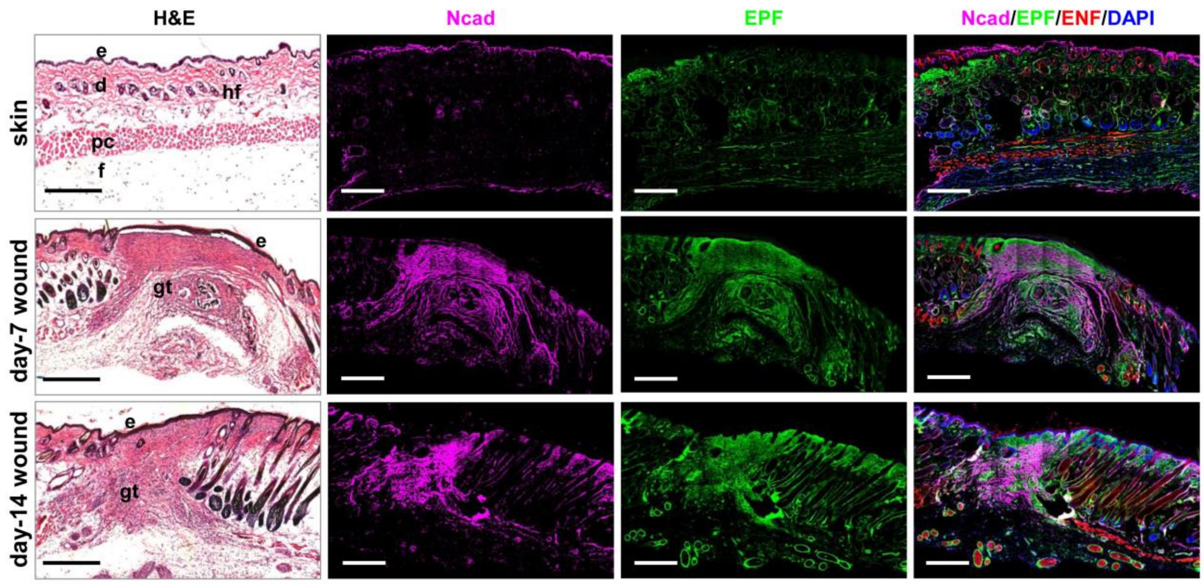

Supplementary Figure 9. N-cadherin expression is elevated in EPFs in physiological wounds.

H&E staining and immunolabeling of N-cadherin (magenta) in skin and *in vivo* wounds at day 7 and day 14 after wounding. EPFs showed in green. e, epidermis; d, dermis; pc, panniculus carnosus; hf, hair follicle; f, fascia; gt, granulation tissue. Scale bars = 500 µm.

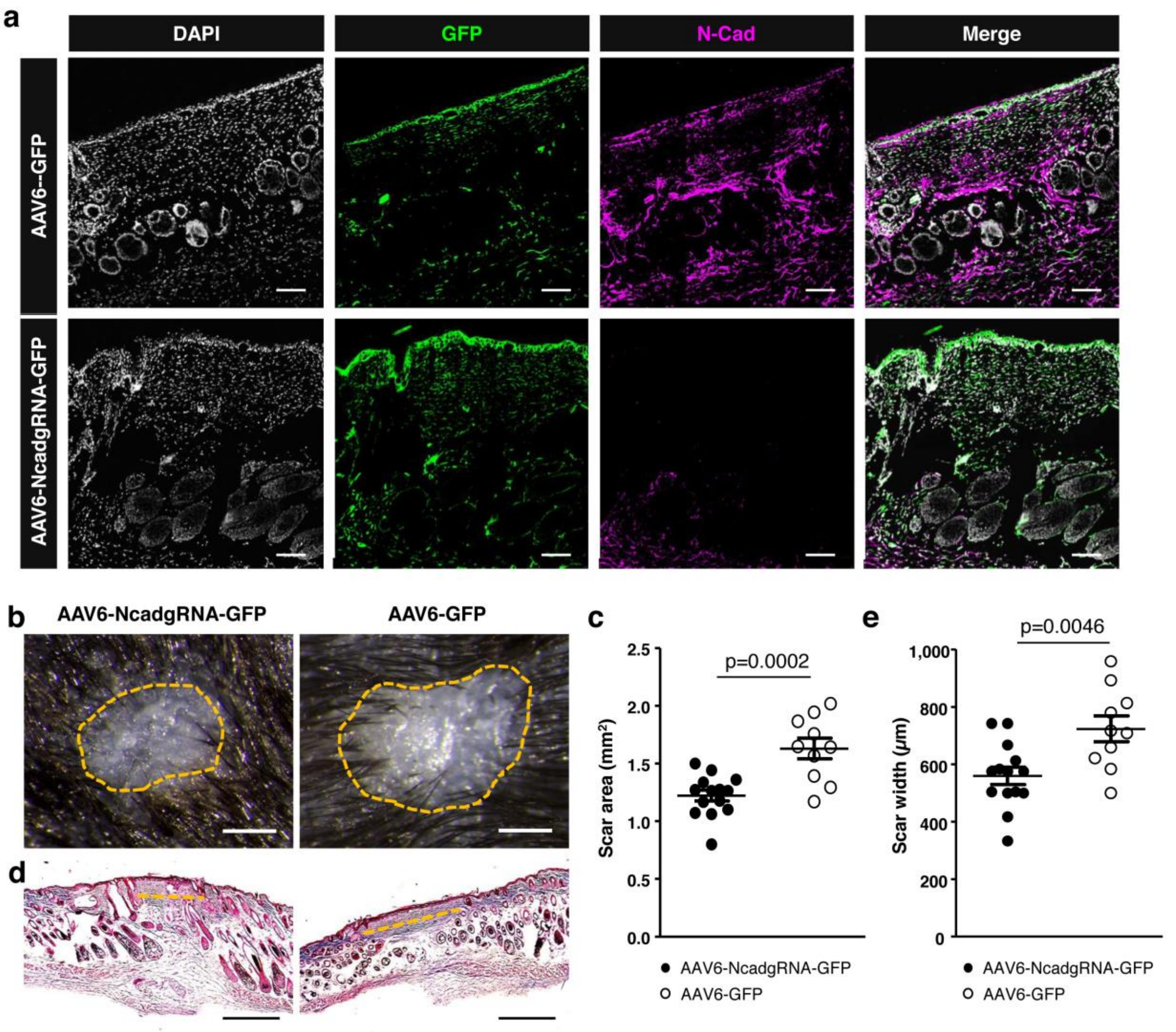

Supplementary Figure 10. Local knockout of N-cadherin reduces scarring with full-body Cas9 mouse model.

Local knockout of N-cadherin in wounds on full-body Cas9 (*R26*$^{\text{Cas9}}$) mice by AAV6 viral particle expressing guide RNA specific to mouse N-cadherin (AAV6-NcadgRNA-GFP). AAV6-GFP transduced wounds served as controls. **a**, immunofluorescence images of scars transduced with AAV6-GFP control virus (upper panel) or with AAV6-NcadgRNA-GFP virus (lower panel), harvested at 14-dpi. **b**, representative stereomicroscopic images of AAV6-NcadgRNA-GFP and AAV6-GFP treated scars at 14-dpi. The yellow dotted lines indicate the scar edges. **c**, quantification of scar area based on histomorphometric analysis. Mean ± SEM, n=14/10, p=0.0002, unpaired two-tailed *t*-test. **d**, Masson's trichrome staining of AAV6-NcadgRNA-GFP and AAV6-GFP treated scars. The orange dotted lines indicate scar width. **e**, quantification of scar width based on histomorphometric analysis. Mean ± SEM, n=14/10, p=0.0046, unpaired two-tailed *t*-test. Scale bars: a = 100 µm; b, d = 500 µm.

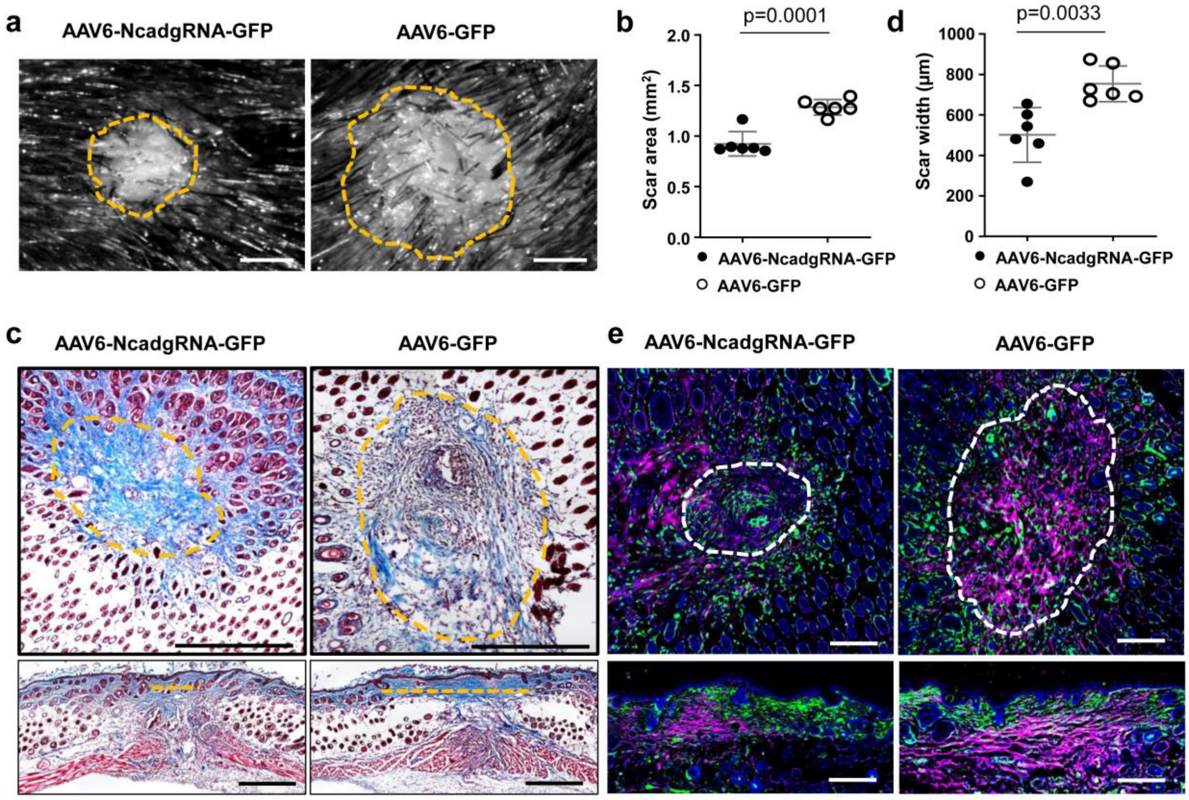

Supplementary Figure 11. Local knockout of N-cadherin in EPFs reduces scarring with Cas9 knock-in mouse model.

Knockout of N-cadherin in EPFs around wounds on $En1^{Cre}R26^{Cas9\text{-}knockin}$ mice by AAV6 viral particle expressing guide RNA specific to mouse N-cadherin (AAV6-NcadgRNA-GFP). AAV6-GFP transduced wounds served as controls. **a**, representative stereomicroscopic images of AAV6-NcadgRNA-GFP and AAV6-GFP treated scars at 14-dpi. The dash lines indicate the scar edge. **b**, quantification of scar area based on histomorphometric analysis. Mean ± SD, n=6, p=0.0001, unpaired two-tailed *t*-test **c**, Masson's trichrome stained transverse (upper panel) and vertical (lower panel) sections from AAV6-NcadgRNA-GFP and AAV6-GFP treated scars. The dash lines indicate scar edge or width. **d**, quantification of scar width based on histomorphometric analysis. Mean ± SD, n=6, p=0.0033, unpaired two-tailed *t*-test. **e**, immunolabeling of N-cadherin on transverse (upper panel) or vertical (lower panel) sections of scars. GFP indicates transduced cells. Dash lines outline the scar edges. Scale bars: 500 µm.
