# Supplementary material for: Injury triggers fascia fibroblast collective cell migration to drive scar formation through N-cadherin
Source: Nat Commun. 2020 Nov 6;11:5653. doi: 10.1038/s41467-020-19425-1 (PMC7648088; doi:10.1038/s41467-020-19425-1)
Supplement: Supplementary file 2 — Description of Additional Supplementary Files [file 41467_2020_19425_MOESM2_ESM.pdf]

Title: Supplementary Movie 1.

Description: Intravital live imaging of in vivo wound at 14-dpi on the back of *En1*^{Cre}*;R26*^{mTmG} mice.

Title: Supplementary Movie 2.

Description: GPF channel (EPFs) in every 10 frames from the intravital live imaging (Supplementary Movie 1). Purple dash lines indicate the predicted cellular orientation of EPFs.

Title: Supplementary Movie 3.

Description: Live imaging of SCAD chimera assay from 0 h to 16 h.

Title: Supplementary Movie 4.

Description: Live imaging of *En1*^{Cre}*;R26*^{mTmG} SCAD from 72 h to 96 h.

Title: Supplementary Movie 5.

Description: Live imaging of mCherry nuclear reporter (*En1*^{Cre}*;R26*$^{LSL\text{-}H2B\text{-}mCherry}$) SCAD from 72 h to 96 h.

Title: Supplementary Movie 6.

Description: Migratory tracks of EPFs in live imaging of *En1*^{Cre}*;R26*$^{LSL\text{-}H2B\text{-}mCherry}$ SCAD from 72 h to 96 h. The colour indicates the time, with blue at the beginning and red at the end of the movie.

Title: Supplementary Movie 7.

Description: Live imaging of mCherry nuclear reporter (*Wnt1*^{Cre}*;R26*$^{LSL\text{-}H2B\text{-}mCherry}$) oral SCAD from 72 h to 96 h.

Title: Supplementary Movie 8.

Description: Migratory tracks of WPFs in live imaging of *Wnt1*^{Cre}*;R26*$^{LSL\text{-}H2B\text{-}mCherry}$ oral SCAD from 72 h to 96 h. The colour indicates the time, with blue at the beginning and red at the end of the movie.

Title: Supplementary Movie 9.

Description: Live imaging of two-colour membrane reporter (*Wnt1*^{Cre}*;R26*^{mTmG}) oral SCAD from 72 h to 96 h.

Title: Supplementary Movie 10.

Description: 3D reconstruction of 500 µg/ml Exherin treated d4 *En1*^{Cre}*;R26*^{mTmG} SCAD with immunolabelling of Collagen I (magenta). EPFs are showed in GFP channel.

Title: Supplementary Movie 11.

Description: Live imaging of 500 µg/ml Exherin treated *En1*^{Cre}*;R26*$^{LSL\text{-}H2B\text{-}mCherry}$ SCAD from 72 h to 96 h.

Title: Supplementary Movie 12.

Description: 3D reconstruction of a punch biopsy of hypertrophic scar from human breast skin with immunolabelling of N-cadherin (magenta). Second harmonic generation signal is showed in cyan.

Title: Supplementary Data 1.

Description: Mass spectrometry dataset of proteins from day-0 and day-5 SCAD, *in vivo* scar, and adjacent skin.
