# Supplementary material for: Injury triggers fascia fibroblast collective cell migration to drive scar formation through N-cadherin
Source: Nat Commun. 2020 Nov 6;11:5653. doi: 10.1038/s41467-020-19425-1 (PMC7648088; doi:10.1038/s41467-020-19425-1)
Supplement: Supplementary file 16 — Reporting Summary [file 41467_2020_19425_MOESM16_ESM.pdf]

nature research

Corresponding author(s): Yuval Rinkevich

Last updated by author(s): Oct 1, 2020

Reporting Summary

Nature Research wishes to improve the reproducibility of the work that we publish. This form provides structure for consistency and transparency in reporting. For further information on Nature Research policies, see our Editorial Policies and the Editorial Policy Checklist.

Statistics

For all statistical analyses, confirm that the following items are present in the figure legend, table legend, main text, or Methods section.

n/a	Confirmed	
☐	☒	The exact sample size (n) for each experimental group/condition, given as a discrete number and unit of measurement
☐	☒	A statement on whether measurements were taken from distinct samples or whether the same sample was measured repeatedly
☐	☒	The statistical test(s) used AND whether they are one- or two-sided<br>*Only common tests should be described solely by name; describe more complex techniques in the Methods section.*
☒	☐	A description of all covariates tested
☒	☐	A description of any assumptions or corrections, such as tests of normality and adjustment for multiple comparisons
☐	☒	A full description of the statistical parameters including central tendency (e.g. means) or other basic estimates (e.g. regression coefficient) AND variation (e.g. standard deviation) or associated estimates of uncertainty (e.g. confidence intervals)
☐	☒	For null hypothesis testing, the test statistic (e.g. F, t, r) with confidence intervals, effect sizes, degrees of freedom and P value noted<br>*Give P values as exact values whenever suitable.*
☒	☐	For Bayesian analysis, information on the choice of priors and Markov chain Monte Carlo settings
☒	☐	For hierarchical and complex designs, identification of the appropriate level for tests and full reporting of outcomes
☒	☐	Estimates of effect sizes (e.g. Cohen's d, Pearson's r), indicating how they were calculated

Our web collection on statistics for biologists contains articles on many of the points above.

Software and code

Policy information about availability of computer code

Data collection	Leica Application Suite v4.8<br>AxioVision (Carl Zeiss)<br>Zen (Carl Zeiss)<br>BD FACSDiva Software v6.1.3
Data analysis	ImageJ v1.47<br>GraphPad Prism v6.0<br>Imaris v9.1.0<br>Summit v4.3

For manuscripts utilizing custom algorithms or software that are central to the research but not yet described in published literature, software must be made available to editors and reviewers. We strongly encourage code deposition in a community repository (e.g. GitHub). See the Nature Research guidelines for submitting code & software for further information.

Data

Policy information about availability of data

All manuscripts must include a data availability statement. This statement should provide the following information, where applicable:

- Accession codes, unique identifiers, or web links for publicly available datasets
- A list of figures that have associated raw data
- A description of any restrictions on data availability

The proteomics data have been deposited to the ProteomeXchange Consortium via the PRIDE partner repository with the dataset identifier PXD016068. The data that support the findings of this study are available from the authors on reasonable request.

Field-specific reporting

Please select the one below that is the best fit for your research. If you are not sure, read the appropriate sections before making your selection.

☒ Life sciences ☐ Behavioural & social sciences ☐ Ecological, evolutionary & environmental sciences

For a reference copy of the document with all sections, see nature.com/documents/nr-reporting-summary-flat.pdf

Life sciences study design

All studies must disclose on these points even when the disclosure is negative.

Sample size	No statistical method was used to predetermine sample size. Required experimental sample sizes were estimated based on previous established protocols in the field. The sample sizes were adequate as the differences between experimental groups were reproducible. All n values are clearly indicated within the figure legends.
Data exclusions	No data was excluded from the analysis.
Replication	All experiments were performed at least three times with similar results. The live imaging data showed in Fig. 1d-g, Fig. 2b-f, Fig. 3f-i, Fig. 6e, g, and extended data figure 5d were generated from one representative video respectively.
Randomization	Mice were randomly divided into treatment groups.
Blinding	No experiments presented in this study required blinding.

Reporting for specific materials, systems and methods

We require information from authors about some types of materials, experimental systems and methods used in many studies. Here, indicate whether each material, system or method listed is relevant to your study. If you are not sure if a list item applies to your research, read the appropriate section before selecting a response.

Materials & experimental systems

n/a	Involved in the study	
☐	☒	Antibodies
☒	☐	Eukaryotic cell lines
☒	☐	Palaeontology and archaeology
☐	☒	Animals and other organisms
☐	☒	Human research participants
☒	☐	Clinical data
☒	☐	Dual use research of concern

Methods

n/a	Involved in the study	
☒	☐	ChIP-seq
☐	☒	Flow cytometry
☒	☐	MRI-based neuroimaging

Antibodies

Antibodies used	Primary antibodies for immunostaining:<br>mouse anti-N-cadherin (Clone GC4, Sigma-Aldricg, C3865, 1:100)<br>goat anti-FSP1 (Abcam, ab58597, 1:100)<br>goat anti-PDGFRα (R&D systems, AF1062, 1:50)<br>rabbit anti-Collagen I (Rockland, 600-401-103-0.1, 1:150)<br>rabbit anti-Collagen III (Abcam, ab7778, 1:150)<br>rabbit polyclonal anti-Fibronectin (Abcam, ab23750, 1:200)<br>rabbit polyclonal anti-αSMA (Abcam, ab5694, 1:100)<br>rat anti-CD45 (clone IBL-3/16, Abcam, ab23910, 1:500)

rabbit anti-CD31 (Novus Biologicals, NB100-2284, 1:500)
rat anti-EpCAM (clone G8.8, Abcam, ab92382, 1:500)
rabbit anti-LYVE1 (Abcam, ab14917, 1:500)
rabbit anti-Myf-5 (Santa Cruz, sc-302, 1:500)
rabbit anti-FABP4 (clone EPR3579, Abcam, ab92501, 1:500)
rabbit anti-Integrin αv (clone EPR16800, Abcam, ab179475, 1:500)
rabbit anti-CTNNA1 (α-catenin) (clone EP1793Y, Abcam, ab51032, 1:500)
rabbit anti-Decorin (Abcam, ab175404, 1:500)
rat anti-THY1(CD90) (Abcam, ab3105, 1:100)
rabbit-anti-DLK1 (Abcam, ab21682, 1:200)
rat-anti-F4/80 (Abcam, ab90247, 1:500)
rat anti-Ki67 (clone SP6, Abcam, ab16667, 1:500)

Seconary antibodies:
PacificBlue-, AlexaFluor488-, AlexaFluor568, or AlexaFluor647-conjugated secondary antibodies against suitable species (Life technologies, 1:500)

Antibodies used for flow cytometry/FACS sorting:
APC-anti-CD45 (BioLegend, 103112, 1:200)
APC-anti-EpCAM (CD326) (BioLegend, 118214, 1:200)
APC-anti-PECAM1(CD31) (BioLegend, 102410, 1:200)
APC-Ter119 (BioLegend, 116212, 1:200)
APC-Tie2 (CD202b) (BioLegend, 124008, 1:200)
eFluor660-anti-LYVE1 (eBioscience, 50-0443-82, 1:200)

Validation	See manufacturers' notes. Antibodies were additionally validated using respective isotype antibodies in immunofluorescence assays.

Animals and other organisms

Policy information about studies involving animals; ARRIVE guidelines recommended for reporting animal research

Laboratory animals	Following mouse strains were used, both males and females, adult at 8-12 weeks old or neonatal at postnatal P0-P2:<br>C57BL/6J wild type<br>En1Cre (En1tm2(cre)Wrst)<br>Wnt1Cre<br>R26mTmG (Gt(ROSA)26Sortm4(ACTB-tdTomato,-EGFP)Luo)<br>ROSA26LSL-H2B-mCherry<br>R26iDTR (Gt(ROSA)26Sortm1(HBEGF)Awai)<br>B6.129S6(SJL)-Cdh2tm1Glr/J<br>B6J.129(B6N)-Gt(ROSA)26Sortm1(CAG-cas9*,-EGFP)Fezh/J<br>Gt(ROSA)26Sortm1(Cas9)Rad
Wild animals	The study did not involve wild animals.
Field-collected samples	The study did not involve samples collected from field.
Ethics oversight	Government of Upper Bavaria , Germany

Note that full information on the approval of the study protocol must also be provided in the manuscript.

Human research participants

Policy information about studies involving human research participants

Population characteristics	Healthy donors between 18 - 65 years of age, both genders, underwent plastic surgeries.
Recruitment	Fresh human skin and scar biopsies, from various anatomic locations, were collected through the Department of Dermatology and Allergology, Klinikum rechts der Isar, Technical University Munich. Informed consent was obtained from all subjects prior to skin biopsies.
Ethics oversight	Ethikkommission der Bayrischen Landesärztekamme, reference number 85/18S.

Note that full information on the approval of the study protocol must also be provided in the manuscript.

Flow Cytometry

Plots

Confirm that:

- [x] The axis labels state the marker and fluorochrome used (e.g. CD4-FITC).
- [x] The axis scales are clearly visible. Include numbers along axes only for bottom left plot of group (a 'group' is an analysis of identical markers).
- [ ] All plots are contour plots with outliers or pseudocolor plots.
- [x] A numerical value for number of cells or percentage (with statistics) is provided.

Methodology

Sample preparation	Fascia were physically separated from the back-skin of En1Cre;R26mTmG mice under the fluorescence stereomicroscope. Harvested tissue was minced with surgical scissors and digested with an enzymatic cocktail containing 1 mg/ml Collagenase IV, 0.5 mg/ml Hyaluronidase, and 25 U/ml DNase I (Sigma-Aldrich) at 37°C for 30 min. The resulted single cell suspension was filtered and incubated with fluorophore conjugated antibodies (dilution 1:200) at 4° C for 30 min. Cells were washed and stained with Sytox blue dye (dilution 1:1000. Life technologies, S34857) for dead cell exclusion.
Instrument	Cells were subjected to flow cytometric analysis or FACS sorting using a FACSAria III (BD Bioscience)
Software	Data was collected using the BD FACSDIVA software (BD Bioscience) and analyzed using Summit software (Cytomation)
Cell population abundance	The purity of sorted cells were determined by flow cytometric analysis of the sorted cells with the same gating strategy as during sorting.
Gating strategy	The single cells were gated base on FSC/SSC and FSC-Area/FSC-width. The viable cells were gated within SytoxBlue negative gate, and fibroblasts were gated within lineage-negative (APC-negative) gate (antibodies against lineage markers are APC-conjugated anti-CD45, anti-Ter119, anti-EpCAM, anti-CD31, anti-Lyve1, and anti-Tie2).

- [x] Tick this box to confirm that a figure exemplifying the gating strategy is provided in the Supplementary Information.
